# New tools for the analysis and validation of cryo-EM maps and atomic models

**DOI:** 10.1107/S2059798318009324

**Published:** 2018-09-03

**Authors:** Pavel V. Afonine, Bruno P. Klaholz, Nigel W. Moriarty, Billy K. Poon, Oleg V. Sobolev, Thomas C. Terwilliger, Paul D. Adams, Alexandre Urzhumtsev

**Affiliations:** a Molecular Biophysics and Integrated Bioimaging Division, Lawrence Berkeley National Laboratory, Berkeley, CA 94720, USA; b Department of Physics and International Centre for Quantum and Molecular Structures, Shanghai University, Shanghai, 200444, People’s Republic of China; c Centre for Integrative Biology, Institut de Génétique et de Biologie Moléculaire et Cellulaire, CNRS–INSERM–UdS, 1 Rue Laurent Fries, BP 10142, 67404 Illkirch, France; d Bioscience Division, Los Alamos National Laboratory, Los Alamos, NM 87545, USA; e New Mexico Consortium, Los Alamos, NM 87544, USA; f Department of Bioengineering, University of California Berkeley, Berkeley, CA 94720, USA; g Faculté des Sciences et Technologies, Université de Lorraine, BP 239, 54506 Vandoeuvre-lès-Nancy, France

**Keywords:** cryo-EM, atomic models, model quality, data quality, validation, resolution

## Abstract

Recent advances in the field of electron cryomicroscopy (cryo-EM) have resulted in a rapidly increasing number of atomic models of biomacromolecules that have been solved using this technique and deposited in the Protein Data Bank and the Electron Microscopy Data Bank. Similar to macromolecular crystallography, validation tools for these models and maps are required. While some of these validation tools may be borrowed from crystallography, new methods specifically designed for cryo-EM validation are required. Here, new computational methods and tools implemented in *PHENIX* are discussed, including *d*_99_ to estimate resolution, *phenix.auto_sharpen* to improve maps and *phenix.mtriage* to analyze cryo-EM maps. It is suggested that cryo-EM half-maps and masks should be deposited to facilitate the evaluation and validation of cryo-EM-derived atomic models and maps. The application of these tools to deposited cryo-EM atomic models and maps is also presented.

## Abstract 2

New methods and *PHENIX* tools for quality assessment of cryo-EM maps, atomic models and model-to-map fitting are presented. Results of systematic application of these tools to high-resolution cryo-EM maps and corresponding atomic models are analyzed and discussed.

## 1. Introduction   

**Figure 1:**
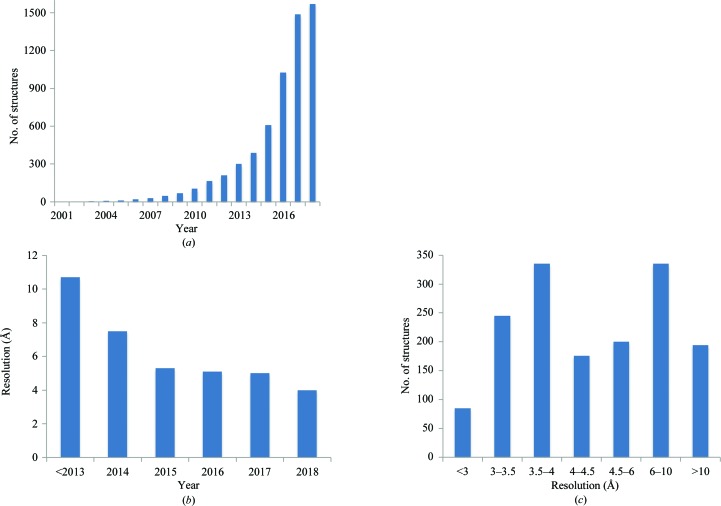
Cryo-EM models in the PDB. (*a*) Cumulative number of models and (*b*) mean resolution extracted from the database by year. (*c*) Distribution of the resolution for all models.

While crystallography is still the predominant method for obtaining the three-dimensional atomic structures of macromolecules, the number of near-atomic resolution structures from electron cryomicroscopy (cryo-EM) is growing exponentially (Fig. 1 ▸; Orlov *et al.*, 2017 ▸). Since the introduction of direct electron detectors (see, for example, Faruqi *et al.*, 2003 ▸; Milazzo *et al.*, 2005 ▸; Deptuch *et al.*, 2007 ▸), cryo-EM is increasingly becoming the method of choice for many macromolecules, particularly since these detectors have been standardized for routine usage. Crystallographic structure determination is a multi-step process that includes sample preparation, obtaining a crystal of the sample, measuring experimental data from that crystal, solving the phase problem and building an atomic model, followed by model refinement and validation (Rupp, 2010 ▸). As an imaging technique, the collection and processing of experimental data is significantly different in structure determination using cryo-EM because there is no phase problem to solve (Frank, 2006 ▸). However, it is very similar to crystallography in the subsequent stages of the process, such as model building, refinement and validation.

It has been widely accepted that model validation (Chen *et al.*, 2010 ▸) is critical in assessing the correctness of a model from chemical, physical and crystallographic viewpoints, which in turn helps to ensure that the result, the atomic model of a structure, is suitable for further uses (see, for example, Read *et al.*, 2011 ▸). Model validation also plays a key role in identifying scientific fraud (Janssen *et al.*, 2007 ▸) and the misinterpretation of experimental data (Chang *et al.*, 2006 ▸; see also Brändén & Jones, 1990 ▸; Kleywegt & Jones, 1995 ▸; Kleywegt, 2000 ▸ and references therein). In crystallography, it took decades for validation methods and tools to become established, mature and gain wide acceptance. Cryo-EM is just entering the era of routine use at near-atomic resolution (Kühlbrandt, 2014 ▸) with atomic models built *de novo* based on experimental maps. While many validation metrics, such as those that assess the geometry of atomic models, can be directly imported from crystallography, others are not readily applicable (such as crystallographic *R* factors). This is mostly because of the nature of the experimental data; for example, there are no experimental structure factor amplitudes in cryo-EM that could be used to calculate *R* factors. To date, there are more than a thousand atomic models in the PDB that were obtained using cryo-EM and that were likely to have been evaluated using tools borrowed from various crystallographic packages or other sources. Thus, an overall quality assessment of these models may be useful (Henderson *et al.*, 2012 ▸; Pintilie *et al.*, 2016 ▸; Joseph *et al.*, 2017 ▸; Neumann *et al.*, 2018 ▸).

Here, we survey cryo-EM maps and derived models as well as discuss tools and methods implemented in the *PHENIX* suite of programs (Adams *et al.*, 2010 ▸) specifically designed to evaluate cryo-EM-derived atomic models and maps. We have used these tools to provide an assessment of the quality of a high-resolution subset (4.5 Å or better) of cryo-EM-derived atomic models that are currently available in the Protein Data Bank (PDB; Bernstein *et al.*, 1977 ▸; Berman *et al.*, 2000 ▸) and the corresponding maps available in the Electron Microscopy Data Bank (EMDB; Lawson *et al.*, 2011 ▸). The analysis shows an improvement in model quality in recent years, while also suggesting that there are opportunities for further improvement that will require the development of new validation tools and procedures.

## 2. Methods   

All of the tools and methods described in this section are either standard *PHENIX* tools or have been implemented in *PHENIX* as part of this work.

### 2.1. Validation   

The aim of modeling experimental data is to find a mathematical description that allows an accurate and unambiguous explanation of the data. This description can then be used to explain known features of the system studied and to predict new features. Subject to validation are the atomic model, the experimental data (three-dimensional reconstruction, in the case of cryo-EM) and the model to data fit. Validating the results of a structural analysis typically requires answering questions such as the following.

(i) How high is my data quality?
(ii) Does my model agree with priors (for example, chemical and physical knowledge)?
(iii) How well does my model fit the experimental data?
(iv) Does my model overinterpret my experimental data? Is my model unique?
(v) What are the method-specific features of the data, model and process of obtaining the model that may affect the quality of the final model? For example, in crystallography, once obtained from data-processing tools, diffraction intensities or amplitudes are never changed or otherwise modified even though the obtained density may depend on phasing with the atomic model under refinement. In contrast, cryo-EM maps may be subjected to various changes [such as masking, focused refinement (von Loeffelholz *et al.*, 2017 ▸), sharpening, blurring *etc.*] throughout the entire process of structure solution; however, once a final map has been obtained it will be constant throughout the atomic model building and refinement process as it is comparable to an independently phased map and thus is model-independent.

Validation normally consists of three components: analysis of the experimental data, analysis of the model and analysis of the fit of the model to the data. These analyses are performed using some well established methods and metrics. Generally, these metrics are of two types: global and local (see, for example, Tickle, 2012 ▸). Global metrics provide concise summaries that are often easy to evaluate (see, for example, Urzhumtseva *et al.*, 2009 ▸); however, they may be misleading as they may not reveal local or low-occurrence violations. For instance, the root-mean-square (r.m.s.) deviation between covalent bond lengths calculated from atomic coordinates of the model and those found in restraints libraries is a global validation metric that is almost universally used in validation reports. While this metric is useful in providing an overall indication of model geometric quality, it is unlikely to reveal one or a few covalent bonds with poor geometry (Morffew & Moss, 1983 ▸; Urzhumtsev, 1992 ▸). In contrast, local metrics, for example the quality of a residue side-chain fit into the density map measured with a map correlation, or validation of (φ, ψ) torsion angles in proteins (Ramachandran *et al.*, 1963 ▸), are good at identifying local issues, but may be voluminous and require careful presentation.

In this work, we only use global validation metrics. While some of these metrics are standard and well documented in the literature, others require explanation, as provided below.

#### 2.1.1. Model–map correlation   

The model–map correlation coefficient [typically referred to as CC, map CC, map correlation or real-space correlation (Brändén & Jones, 1990 ▸; Jones *et al.*, 1991 ▸; see also the overview in Tickle, 2012 ▸, and references therein)] is a metric that shows how well the model fits the map. It is worth noting, though, that map correlation coefficients can sometimes be misleading (Urzhumtsev *et al.*, 2014 ▸). Calculation of the model–map CC requires (i) choosing the CC formula, (ii) obtaining a model-based map and (iii) defining the region of the map to be used to calculate the CC. To make the interpretation of CC values meaningful these three items need to be clearly defined.

*CC calculation*. The CC value between two maps, ρ_1_(**n**) and ρ_2_(**n**), available on the same grid {**n**} may be calculated in two ways. The first method simply calculates the normalized product of densities in the two maps. This calculation is affected by offsetting all values in one or both maps by a constant. The second method calculates the correlation in the same way as the first except that it adjusts each map so that the mean is zero. In this way, the second calculation reflects the covariation of the two maps and is unaffected by offsets in either. The two calculations are
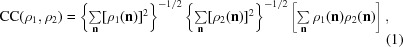
or
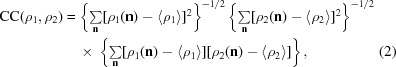
(Joseph *et al.*, 2017 ▸), where 〈〉 indicates an average over all grid points {**n**}. Typically, crystallographic maps have zero mean value and are calculated for the entire unit cell, resulting in no difference between the use of (1) or (2). Cryo-EM maps are not necessarily expected to have a mean of zero (about 70% of maps in the EMDB have a nonzero mean value). Also, they are frequently calculated locally for a subset of the full box containing the image of a molecule. In such cases the formulae (1) or (2) will produce different results. *PHENIX* uses formula (2), *i.e.* the normalized version.

*Model map*. The model map is sampled on the same grid as the experimental map. The use of electron form factors (Peng *et al.*, 1996 ▸; Peng, 1998 ▸; Yonekura *et al.*, 2018 ▸) is essential for the calculated model map to adequately represent the experimental map (Wang & Moore, 2017 ▸; Hryc *et al.*, 2017 ▸). Atomic model parameters such as coordinates, occupancies, atomic displacement parameters (ADPs) and chemical atom types are required for this calculation and are extracted from the input model file (PDB or mmCIF). The parameters of the reconstructed map, which are known as unit-cell parameters in crystallo­graphy, are also required. A complete set of Fourier coefficients to the resolution of the experimental map (see §[Sec sec2.1.2]2.1.2) is calculated.^1^ Finally, the model map is obtained as a Fourier transform of these model Fourier coefficients. There are some technical parameters involved in this process that may vary between implementations in different programs (see, for example, Grosse-Kunstleve *et al.*, 2004 ▸; Afonine & Urzhumtsev, 2004 ▸ and references therein). Also, other approaches exist for obtaining a map from a model (see, for example, Diamond, 1971 ▸; Chapman, 1995 ▸; Sorzano *et al.*, 2015 ▸).

*Map region for the CC calculation*. Depending on the question at hand, different regions of the map, *i.e.* different sets of {**n**} in (1) or (2), may be used to calculate the correlation coefficient (for example, the entire map or a map masked around the model).

In this work, we analyze several types of real-space correlation coefficients, with each one probing different aspects of the model-to-map fit (Appendix *A* [App appa]). CC_box_ uses the entire map as provided to calculate the CC value; this map may correspond to the whole molecule or a portion carved out as a box around selected atoms. CC_mask_ only uses map values inside a mask calculated around the macromolecule, as described by Jiang & Brünger (1994 ▸). CC_volume_ and CC_peaks_ only compare the map regions with the highest density values. Intuitively, they are related to the atom-inclusion score (Lunina & Lunin, personal communication; Pintilie & Chiu, 2012 ▸) and to how maps are inspected visually on graphical displays: typically maps are inspected above a certain contouring threshold level, while regions below this level are ignored. For CC_volume_ calculations the region is defined by the *N* highest value points in the model-calculated map, with *N* being the number of grid points inside the molecular mask (which refers to the molecular volume). CC_peaks_ uses the union of regions defined by the *N* highest value points in the model-calculated map and the *N* highest value points in the experimental map. In the following, we show that these correlation coefficients provide redundant information, with only three of them being required to capture the unique features of the model-to-map fit.

*Map–model correlation in Fourier space*. Model-to-map fit can also be evaluated in Fourier space by calculating the correlation between Fourier map coefficients binned in resolution shells. The calculated CC values are typically represented as a function of the inverse of resolution and are called the Fourier shell correlation (FSC). The details of FSC calculation can be complicated and are not always well defined, as masking may be carried out as part of the process (Harauz & van Heel, 1986 ▸; see also van Heel *et al.*, 1982 ▸; Saxton & Baumeister, 1982 ▸; van Heel, 1987 ▸; Rosenthal & Henderson, 2003 ▸; van Heel & Schatz, 2005 ▸; Penczek, 2010 ▸). The details of FSC calculations in this work are described in Appendix *A* [App appa]. The FSC values can be calculated either with the whole map or with one of the half-maps (maps reconstructed independently each using half of the experimental data) depending on the specific goal (see, for example, DiMaio *et al.*, 2009 ▸; Brown *et al.*, 2015 ▸). The FSC curve has a characteristic shape, the intersection of which with a threshold (0.143 or 0.5; Rosenthal & Henderson, 2003 ▸; van Heel & Schatz, 2005 ▸) provides the *d*_FSC_ value used nowadays; however, alternative interpretations exist (van Heel & Schatz, 2017 ▸; Afanasyev *et al.*, 2017 ▸).

#### 2.1.2. Data resolution   

In spite of recent work devoted to a better definition of ‘resolution’ in crystallography and cryo-EM [Rosenthal & Henderson, 2003 ▸; Heymann & Belnap, 2007 ▸; Penczek, 2010 ▸; Evans & Murshudov, 2013 ▸; Karplus & Diederichs, 2012 ▸; Urzhumtseva *et al.*, 2013 ▸; Chen *et al.*, 2013 ▸; Kucukelbir *et al.*, 2014 ▸; see also the web service provided by GlobalPhasing (http://staraniso.globalphasing.org/staraniso_about.html)], there is still debate about the appropriate definition and some confusion, mostly owing to the use of the same term *resolution* for different concepts. This can lead to the misinterpretation of statistics that are not expected to be comparable (see Wlodawer & Dauter, 2017 ▸; Chiu *et al.*, 2017 ▸). Below, we discuss some relevant issues.

The overall resolution reported for cryo-EM maps is typically the *d*_FSC_ obtained using an FSC curve calculated between two half-maps. In cryo-EM, the resolution estimated from the FSC is defined as the maximum spatial frequency at which the information content can be considered to be reliable. This resolution is unrelated to the resolution in the optical sense, which allows the visualization of specific details (Penczek, 2010 ▸). This is one of the first areas of confusion when considering *resolution* in either the cryo-EM or crystallographic contexts. Typically, crystallographic resolution (a high-resolution cutoff of the diffraction data set) is related to the map detail, while *d*_FSC_ is related but in a less straightforward manner (see, for example, the discussions in Malhotra *et al.*, 1998 ▸; Liao & Frank, 2010 ▸).

It is worth noting that a single number is unlikely to be adequate in quantifying the resolution of a three-dimensional cryo-EM image. The notion of local resolution has been introduced for cryo-EM maps (Cardone *et al.*, 2013 ▸; Kucukelbir *et al.*, 2014 ▸), which reports on the spatial variability in the resolution of three-dimensional EM reconstructions. However, much like in crystallography, a single-number estimate of effective resolution in the map, the average resolution, will always be desirable and is likely to be demanded by the community.

*Reported resolution*. Since both the atomic model file and the metadata associated with the corresponding map file typically report the resolution, matching the two resolution values extracted from these two sources is the most simple and naive consistency check. Obviously, the two values are expected to be similar. Furthermore, if half-maps are available then the resolution can be calculated from the FSC curve and compared with the values associated with the deposited model and map files.

*Resolution estimate using atomic model*. If an atomic model corresponding to the experimental map is reasonably placed and refined into the map, an alternative method for estimating the map resolution is possible. In this case, one can pose the question: ‘at what resolution limit is the model-calculated Fourier map most similar to the experimental map?’. The resolution, *d*_model_, of the model-calculated map that maximizes this similarity can be an estimate for the resolution of the experimental map (Appendix *B* [App appb]). Intuitively, this method is expected to be most reliable when the model has been optimized to fit the map well; however, the application of this approach to deposited cryo-EM maps (§[Sec sec3.6.2]3.6.2) does not show a strong dependence on this condition.

Yet another approach to estimate the resolution to which the data contain useful signal is to compute the FSC between the atomic model and experimental map (see Appendix *A* [App appa] for details) and note the point where the FSC approaches 0.5 (Rosenthal & Henderson, 2003 ▸; Rosenthal & Rubinstein, 2015 ▸) or another threshold of choice. We refer to this point as *d*_FSC_model_. Here, we refer to the FSC calculated with respect to the full map calculated with all data.

*Resolution and map detail*. A resolution estimate that is related to the map details may be obtained using the following rationale. One can calculate a Fourier transform of the map and then ask the question: ‘how many of the highest resolution Fourier map coefficients can be omitted before the corresponding real-space map changes significantly?’ This is based on two fundamental facts. Firstly, a Fourier transform of a cryo-EM map defined on a regular grid inside a box corresponds to a box of complex Fourier map coefficients that is an exact Fourier space equivalent of the corresponding real-space map. Secondly, the highest resolution coefficients, which are located towards the corners of the box in Fourier space, may or may not contribute significantly to the map. Gradually removing these highest resolution coefficients, resolution layer by resolution layer, we note the resolution threshold, which we refer to as *d*_99_ (see Appendix *C* [App appc] for details), at which the map calculated without these coefficients starts to differ from the original map; this threshold can be considered to report on the detail in the map.

We developed a procedure to calculate the *d*_99_ value (Appendix *C* [App appc]) and compared it with *d*_FSC_ for all cryo-EM maps extracted from the EMDB; §[Sec sec3.6.3]3.6.3 reports the results.

### 2.2. Extraction of atomic models and maps from the PDB and EMDB   

Atomic models and maps were automatically extracted from the PDB and the EMDB, respectively, to provide matching pairs (model, map). A Python script based on *cctbx* (Grosse-Kunstleve & Adams, 2002 ▸) was written for this purpose. Entries were rejected if any of the items below were true.

(i) The box information (for example, the CRYST1 record in the PDB coordinate file) was impossible to interpret unambiguously considering both the model file and the data associated with the map file.
(ii) MTRIX or BIOMT matrices are present but cannot be extracted owing to syntactical errors in the records, or the corresponding matrices do not satisfy the numerical requirements for rotation matrices.
(iii) The model or map contains errors such as a C^β^ atom in a Gly residue.
(iv) The file is not accessible (for example, public release placed on hold).
(v) The file contains multiple models.
(vi) The model mostly consists of single-atom residues (such as C^α^ or P-only models).
(vii) Half-maps were rejected because the gridding did not match the gridding of the full map.

A total of 1548 model–map pairs were extracted (1488 unique model files), with 194 entries having half-maps available. For all partial models, as indicated by MTRIX or BIOMT records, full models were generated and used in the calculations described below.

For analysis of model geometry and model-to-map fit, only entries with a resolution of 4.5 Å or better were used. This is because this resolution range allows atomic models to be a robust tool for the interpretation of density maps (for example, protein side chains can still be seen; Barad *et al.*, 2015 ▸) and also represents the models and maps obtained in recent years.

For analysis of maps and the development of various resolution measures, we used maps with a resolution of 6.0 Å or better (to account for possible map sharpening that can potentially increase the effective resolution).

### 2.3. Tools   

All calculations were performed fully automatically, with no manual intervention, and therefore can be routinely repeated. Tools available in *PHENIX* [*MolProbity* (Chen *et al.*, 2010 ▸) and *EM-Ringer* (Barad *et al.*, 2015 ▸)] were used to calculate various statistics such as Ramachandran plots, residue side-chain rotamer outliers and model–map correlations. The *cctbx* software library was used to extract files from databases and to compute, process and accumulate statistics. Some new tools were developed to address specific tasks (for example, *phenix.mtriage* to analyze cryo-EM maps). All scripts used in this work are publicly available (http://phenix-online.org/phenix_data/afonine/cryoem_validation/). *PyMOL* (DeLano, 2002 ▸) and *UCSF Chimera* (Goddard *et al.*, 2018 ▸) were used for molecular graphics.

## 3. Results and discussion   

This section summarizes the results of the application of the above-described validation tools to models and maps extracted from the PDB and EMDB.

### 3.1. Model geometry   

The topic of atomic model validation for crystallographic and cryo-EM-derived models has been discussed at some length in reports from wwPDB-convened task forces (see, for example, Henderson *et al.*, 2012 ▸). Here, we briefly summarize some of the salient points and provide some additional details.

It is widely recognized that acceptable r.m.s. deviations for covalent bonds and angles from the refinement restraint targets should not exceed approximately 0.02 Å and 2.5°, respectively (see, for example, Jaskolski *et al.*, 2007*a* ▸; Wlodawer *et al.*, 2008 ▸, and references therein). These rule-of-thumb-based target values may be larger for models derived using very high-resolution data because such data may be able to provide experimental evidence that supports larger deviations. Inversely, they are expected to be lower in case of low-resolution data because these data cannot readily support such deviations (Jaskolski *et al.*, 2007*a* ▸,*b* ▸; Stec, 2007 ▸; Tickle, 2007 ▸; Karplus *et al.*, 2008 ▸).

Ramachandran and rotamer outliers, as well as C^β^ deviations, are assessed statistically based on the examination of many high-quality models solved and refined against high-resolution crystallographic data (Chen *et al.*, 2010 ▸). Some conformations may be labeled as outliers not because a particular rotameric state or combination of (φ, ψ) angles is impossible, but because it is found to be uncommon based on the analysis of a large number of high-quality structures. Therefore, an *outlier* does not necessarily mean *incorrect*, but rather something that needs to be investigated and justified by the experimental data. An example of a Ramachandran plot outlier that in fact is valid can be found in isocyanide hydrat­ase (PDB entry 3NoQ 2; Lakshminarasimhan *et al.*, 2010 ▸). A valid outlier must be supported by the experimental data (unambiguously resolved in the map, for instance) and be justified by local chemistry (for example, a strained conformation stabilized by hydrogen bonding). The overall data resolution is neither the only nor the most important resolving factor of the data. Other factors, such as data completeness in crystallography or local variations of resolution in cryo-EM, may be equally important. With this in mind, it will be increasingly unlikely that outliers can be supported by the experimental data as the resolution worsens. In most cases we would expect that a model refined against data at a resolution of ∼3 Å or worse would have very few or no justifiable geometric outliers.

The *MolProbity* clashscore (Chen *et al.*, 2010 ▸) is a measure of unfavorable steric clashes between atoms in the model. The lower the clashscore values the better, and high-quality models are expected to have a minimal number of clashes and no overlapping atoms.

**Figure 2:**
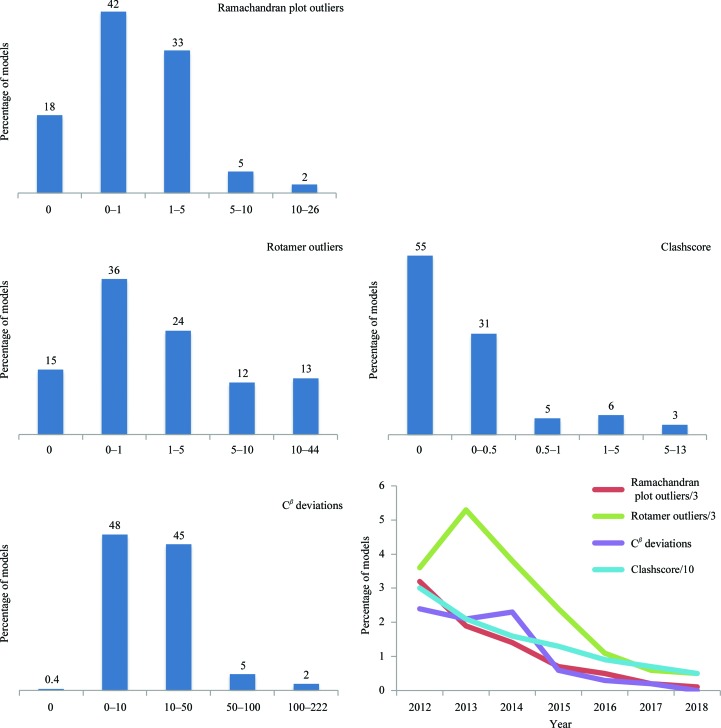
Model-geometry metrics for models at 4.5 Å resolution or better. The number at the top of each bar shows the percentage of structures that fall into the category. *x* axis: percentages of outliers (rotamer, Ramachandran and C^β^ deviation) and clashscore value. Curves show by-year average percentages of Ramachandran, rotamer and C^β^ deviation outliers, as well as values of clashscore. For clarity in presentation, the percentages of rotamer and Ramachandran plot outliers are scaled by 1/3 and the clashscore is scaled by 1/10.

Fig. 2 ▸ shows a summary of the geometry-validation metrics used in this study and calculated for all considered PDB/EMDB models. While the overall number of models having severe geometric violations is rather substantial, the yearly statistics show steadily improving model-geometry quality.

### 3.2. Secondary-structure annotation   

Information about protein secondary structure (SS) has many uses, ranging from structural classification and tertiary-structure prediction to aiding in multiple sequence alignment. One example where SS information is particularly important is atomic model refinement against low-resolution data (crystallographic or cryo-EM) that are typically insufficient to maintain a reasonable geometry in secondary-structure elements during refinement. Therefore, specific restraints on secondary-structure elements (Headd *et al.*, 2012 ▸) can be generated using the SS annotation encoded in the HELIX and SHEET records of model files or calculated dynamically by refinement software. The latter can be problematic since the input model may not be of sufficient quality to reliably derive the correct SS annotation. Therefore, it is desirable that validated SS information be provided and used for these purposes.

Each SS record unambiguously defines its type (for example helix or sheet), which in turn defines the hydrogen-bond pattern and expected region of the Ramachandran plot for the corresponding residues. The information derived from the SS annotations can then be matched against the information calculated from the atomic model. This provides a way to validate the consistency of SS annotations with the deposited atomic model. *phenix.secondary_structure_validation* is a *PHENIX* tool that is designed to perform this validation.

**Figure 3:**
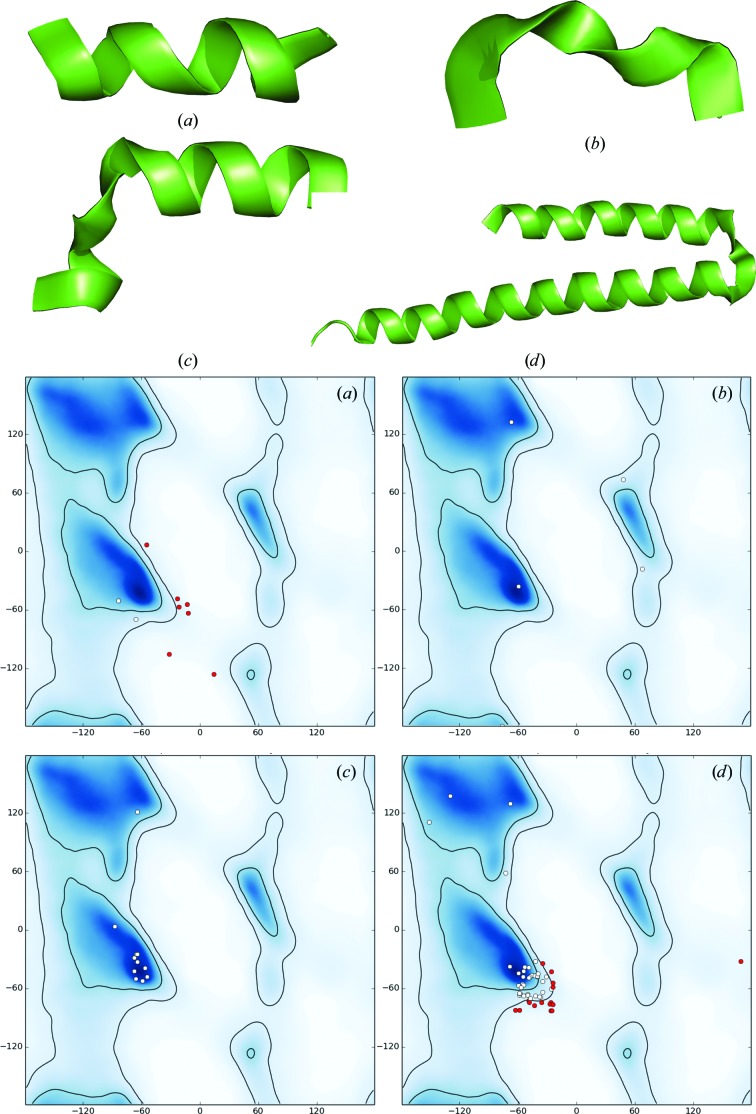
Examples of problematic secondary-structure (SS) annotations shown as pairs of cartoon representation and corresponding Ramachandran plot. (*a*) The α-helix looks plausible although slightly distorted, but most residues are Ramachandran plot outliers. (*b*) The α-helix is obviously distorted; there are no Ramachandran plot outliers, but only one angle belongs to the α-­helix region of the plot. (*c*) Distorted α-helix with all but one residue belonging to the expected Ramachandran plot region. (*d*) Apparently two α-helices annotated as one with many (φ, ψ) pairs being out of the α-helix region.

Of the cryo-EM models considered in this analysis that contain secondary-structure annotations, 47% have at least one Ramachandran plot outlier within an annotated secondary-structure element, 76% have at least one residue with a mismatch between the annotation and actual (φ, ψ) angles (for example, a residue that is annotated as belonging to HELIX but in fact belongs to a β region of the plot) and 99% of models have at least one hydrogen bond defined by provided annotation that is longer than 3.5 Å (calculations performed by the *phenix.secondary_structure_validation* tool). Fig. 3 ▸ illustrates some typical situations.

### 3.3. Model-to-data fit   

**Figure 4:**
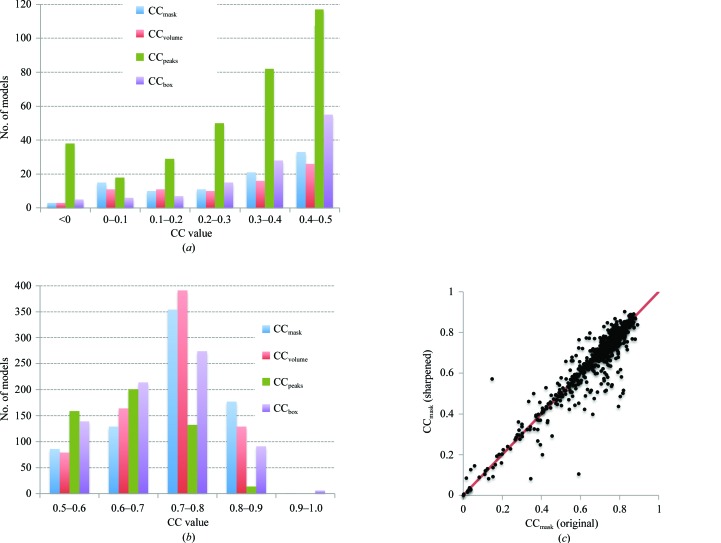
Distribution of all four correlation measures (CCs) considered in this work, CC_box_, CC_mask_, CC_volume_ and CC_peaks_, for models at 4.5 Å resolution or better; values (*a*) below 0.5 and (*b*) above 0.5 are shown separately for clarity. (*c*) Comparison of CC_mask_ calculated using the original maps and the same maps sharpened with *phenix.auto_sharpen* (resolution 4.5 Å or better). The overall CC_mask_ averages are 0.676 and 0.665 using the original and sharpened maps, respectively.

To quantify the model-to-map fit, we calculated correlation coefficients between the model and corresponding experimental maps as described in §[Sec sec2.2]2.2 and Appendix *A* [App appa]. Figs. 4 ▸(*a*) and 4 ▸(*b*) show the distribution of these CC values. For about 40% of the deposited models, at least one of these correlation coefficients is below the value of 0.5 which may be considered as a low correlation (Appendix *E* [App appe]). Several scenarios can be envisaged leading to substantially different values for the various CC measures. For example, a partial model (say, one chain of a symmetric molecule) may perfectly fit the map, leading to a high CC_mask_, while such a model obviously does not explain the whole map, resulting in CC_peaks_ being low. Conversely, a poorly fitting model with low CC_mask_ may be placed into a large box, making CC_box_ higher. There may be a number of plausible mixtures of these scenarios where only selected CC metrics would indicate problems. This supports the simultaneous use of several types of correlation coefficients, with each one being suited for identifying specific problems. In the following, we attempt to determine which of the CC metrics are necessary.

**Figure 5:**
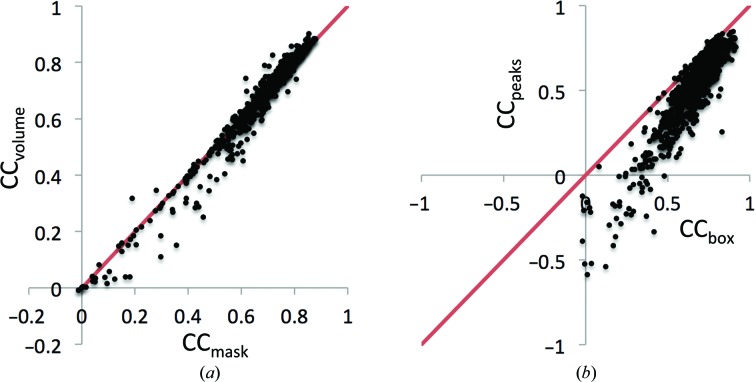
Distribution of CC_mask_ *versus* CC_volume_ (*a*) and CC_box_ *versus* CC_peaks_ (*b*) for entries at a resolution of 4.5 Å or better.

For structures determined at higher resolutions, a molecular envelope extracted from a map is expected to be similar to the envelope built from the model following Jiang & Brünger (1994 ▸). Consequently, the values of CC_volume_ and CC_mask_ are expected to be similar (Fig. 5 ▸ *a*). However, this is not the case when the structure contains mixtures of well and less well defined parts; an example is PDB entry 3JBS.^3^ Therefore, the CC_volume_ and CC_mask_ values and the difference between them may be indicative of a variability in model quality within a structure.

As opposed to CC_mask_ and CC_volume_, two other coefficients, CC_box_ and CC_peaks_, quantify the fit of a given model against the entire map and both indicate the presence of non-interpreted parts of the map. An advantage of CC_peaks_ over CC_box_ is its independence of box size, while CC_box_ depends on the size of the box. Calculation of CC_box_ includes the comparison of two relatively flat regions outside the structure that artificially results in larger values, CC_box_ ≥ CC_peaks,_ for all models (Fig. 5 ▸ *b*). Consequently, any model with a particular value of CC_peaks_ automatically has a value of CC_box_ that is at least as large.

In conclusion, the triplet of correlation coefficients CC_volume_, CC_mask_ and CC_peaks_ are nonredundant and comprise the set of CCs that should be used to quantify the overall quality of the model-to-map fit.

**Figure 6:**
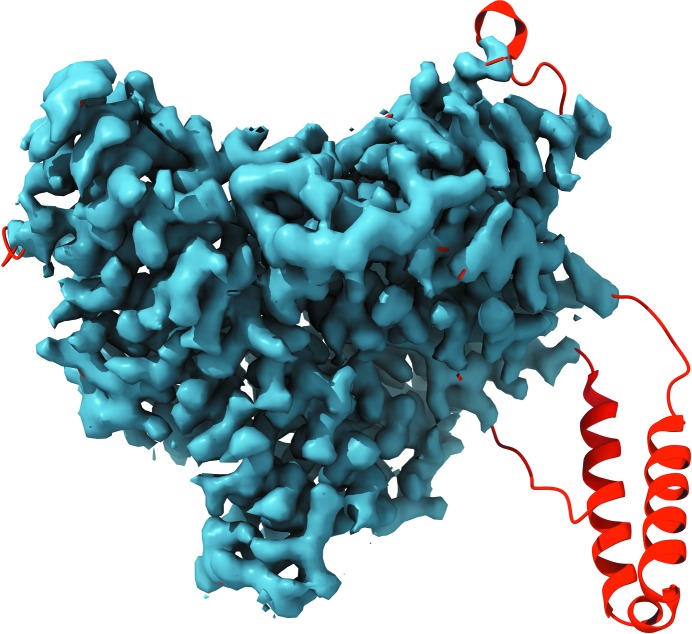
Model and map (PDB and EMDB codes 3J9E and 6240, respectively; resolution 3.3 Å) showing some parts of the model that do not fit the map at any chosen threshold contouring level (shown in red).

**Figure 7:**
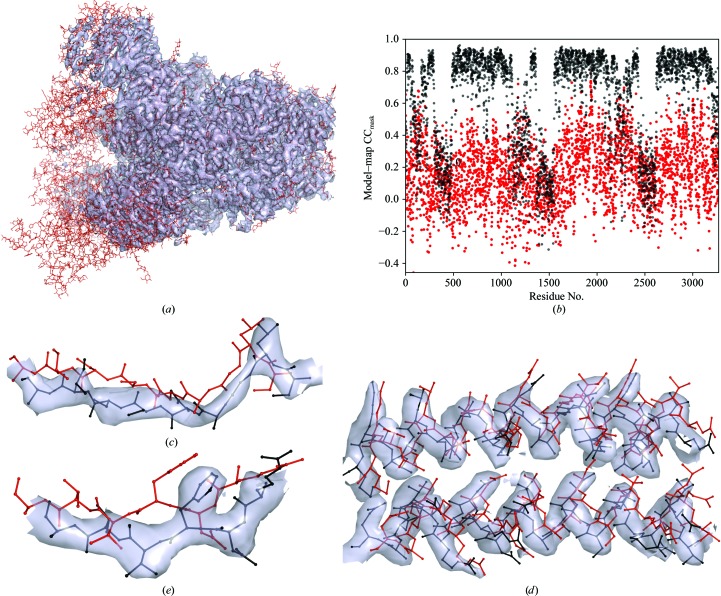
Model and map (PDB and EMDB codes 6CRZ and 7577, respectively; resolution 3.3 Å) showing a combination of two issues. (*a*) Some parts of the model do not fit the map. (*c*, *d*, *e*) Improvements that can be achieved after a round of refinement using *phenix.real_space_refine*: compare the model-to-map fit before (red) and after (black) refinement. (*b*) Model–map correlation CC_mask_ shown per residue: red and black are before and after refinement, respectively.

Finding about 40% of the models with values of CC_volume_, CC_mask_ or CC_peaks_ below an arbitrary but plausible threshold of 0.5 suggests that the fit of the model to the map could be improved. A possible reason for such rather low CC values for the deposited structures could be that sharpened maps might have been used to obtain these models but these maps were not deposited. Using sharpened maps to calculate CC_mask_ (Fig. 4 ▸ *c*) did not change the correlation coefficients substantially: the CC_mask_ values using sharpened maps are similar but slightly lower overall compared with using the original maps. An alternative hypothesis is an incomplete optimization of the model parameters (coordinates, occupancies of ADPs) with respect to the map. Indeed, as discussed below in §[Sec sec3.4]3.4, we find that about 31% of all models examined possess unrealistic occupancy or/and ADP values, such as all being set to zero or other unlikely values. Given that occupancies and ADPs are used to calculate the model maps (see §[Sec sec2.1.1]2.1.1), it is not surprising to find low CC values for such models. Figs. 6 ▸, 7 ▸ and 8 ▸ serve as examples of cases in which incomplete optimization can result in low model-to-map correlation and show that rather simple refinement can address some of the issues (Figs. 7 ▸ and 8 ▸). Finally, some extremely low model–map correlations (*e.g.* CC < 0.1; Fig. 4 ▸ *a*) can be explained by origin mismatch between the map and model (for example, PDB entry 3A5X and EMDB entry 1641).

### 3.4. Atomic displacement parameters and occupancy factors   

Atomic displacement parameters (ADPs) and occupancies are key parameters required to calculate a model-based map. The use of this map may range from an assessment of the fit of the model to the data using the various CCs described earlier to a refinement in which the model is improved by optimizing the fit of the model-calculated map to the experimental map. Therefore, the correctness of both occupancy and ADP values is important. As part of our analysis, we found 18 models with more than 1% of the atoms having zero occupancy. About 246 models have atoms with ADP values less than 0.01. Overall, about 31% of models possess occupancies or ADPs that are unlikely to be realistic. These cases are likely to contribute to low model-to-map correlation (Fig. 4 ▸).

### 3.5. Assessment of local residue fit in high-resolution models with *EM-Ringer*   

*EM-Ringer* is an extension of the *Ringer* method (Lang *et al.*, 2010 ▸, 2014 ▸) that has been developed for cryo-EM models and maps (Barad *et al.*, 2015 ▸). The method assesses the quality of the atomic model by calculating the local fit of the amino-acid residue side chain to the map in light of the rotameric state of the residue. Mismatches between the peaks in density around a side-chain position and its valid rotameric states are interpreted as a problem with the placement of the residue. The scores for individual residues are aggregated into a single number: the *EM-Ringer* score. A high score is better, with better than 1.5 being desirable, while a score below 1 is very poor. More than half of these models at a resolution of 4 Å or better have *EM-Ringer* scores above 1.5, while about a third of them have a score below 1, suggesting potential problems with the placement of the side chains in these models.

### 3.6. Data resolution   

#### 3.6.1. Resolution recalculated from half-maps   

The most trivial assessment of resolution is a consistency check between the value reported for the deposited model (for example, extracted from a PDB or mmCIF file) and that associated with the corresponding map in the EMDB. One would expect that the values should match exactly or at least very closely. We find that for about 27% of entries the reported resolution values do not match. Typographical errors during deposition may be responsible for some of these discrepancies, but others are less easy to understand.

**Figure 9:**
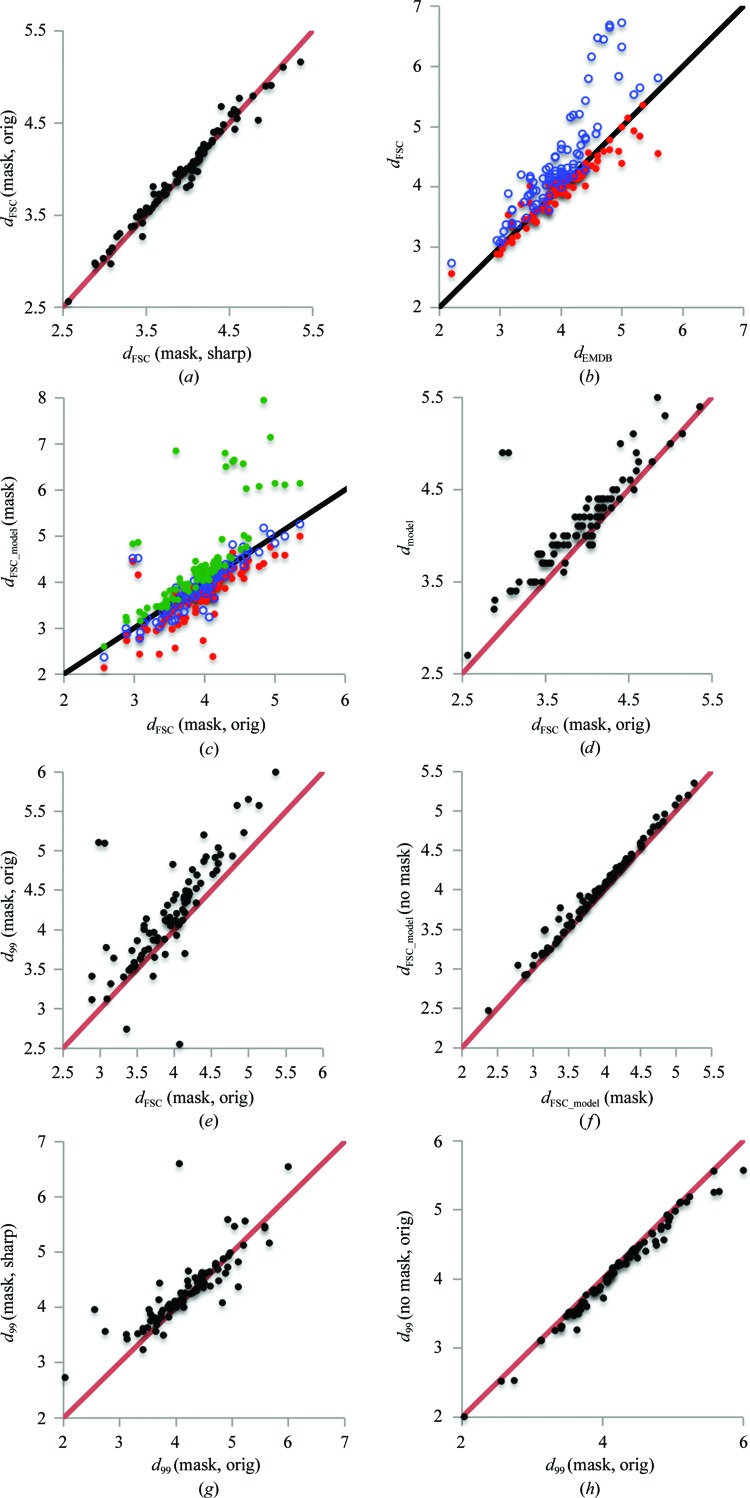
Scatter plots showing the relationship between the different resolution estimates and their different ways of calculation. (*a*) *d*_FSC_ calculated using original half-maps *versus* *d*_FSC_ using sharpened half-maps; a mask was used in both cases. As expected, *d*_FSC_ is essentially insensitive to map sharpening. (*b*) Comparison of *d*_FSC_ extracted from the EMDB (referred to as *d*_EMDB_) with recalculated values using available half-maps with masking applied (red) and not applied (blue); no sharpening was used in both cases. (*c*) *d*_FSC_model_ calculated at FSC 0 (red), 0.143 (blue) and 0.5 (green) *versus d*_FSC_ from available half-maps (using a mask, no sharpening). The correlation CC(*d*_FSC_, *d*_FSC_model_) is 0.929, 0.959 and 0.973 for FSC thresholds at 0.5, 0 and 0.143, respectively. (*d*) *d*_model_ *versus* *d*_FSC_ calculated using original half-maps (no sharpening). The correlation is rather marked, but clearly *d*_model_ shows lower resolution, likely owing to smearing by atomic displacement parameters. (*e*) *d*_99_ calculated using the original (no sharpening) masked map *versus* *d*_FSC_ calculated using the original half-maps (no sharpening). (*f*) *d*_FSC_model_ calculated with and without masking (taken at FSC = 0.143). Clearly, this resolution metric is not sensitive to using a mask. (*g*) *d*_99_ calculated using original and sharpened maps (masking was used in both cases). Since map attenuation performed using *phenix.auto_sharpen* can sharpen or blur the map, the *d*_99_ value can be smaller or larger, depending on whether blurring or sharpening occurred. (*h*) *d*_99_ calculated using a masked map and an unmasked map (no sharpening in both cases). Since masking eliminates the noise outside the molecular region, *d*_99_ calculated without masking results in systematically smaller values.

Naively, one might expect that a superior approach to assessing the reported resolution would be to recalculate it using the half-maps. In theory, all that is needed for this is two half-maps. The FSC between the two maps can be calculated as described in Appendix *A* [App appa], and the resolution can then be assigned at the point where the FSC drops below 0.143 (Rosenthal & Henderson, 2003 ▸). This is problematic, though. Firstly, only about 10% of cryo-EM entries have half-maps available. Secondly, in practice some masking is typically applied to the map before Fourier coefficient calculation and this may have an impact on the resulting values (Penczek, 2010 ▸; Pintilie *et al.*, 2016 ▸). A more detailed mask is likely to result in a higher resolution estimate. An overly detailed mask may even result in an artificial increase in FSC at high resolution (van Heel & Schatz, 2005 ▸). Given the variety of ways of defining and calculating this mask, it may be difficult to reproduce the published resolution values exactly without knowledge of the original mask. We suggest a simple and easy-to-reproduce way to generate and apply a ‘soft mask’ as described in Appendix *A* [App appa]. Fig. 9 ▸ shows a summary of the resolution metrics considered in this work. Fig. 9 ▸(*a*) proves the known fact that map manipulations such as sharpening do not affect the *d*_FSC_ value significantly. Clearly, for the majority of structures the recalculated values of *d*_FSC_ match the published values (Fig. 9 ▸ *b*), and as the figure shows, masking is important.

A possible reason for the larger deviations in resolution estimates for some structures (Fig. 9 ▸ *b*, some of the red dots further off the diagonal) is the use of masks significantly different from those that we calculate here. To reduce this uncertainty and make the reported results more reproducible and therefore possible to validate (and also to address the problems of model bias and overfitting; discussed in §§[Sec sec3.7]3.7 and [Sec sec3.8]3.8), we second the previous suggestion by Rosenthal & Rubinstein (2015 ▸) that the ‘soft mask’ used should be deposited along with the full and half-maps, with all maps and the mask being defined on the same grid, in the same ‘box’ and with the same origin.

#### 3.6.2. Resolution estimates using deposited models   

Provided that a complete and well refined atomic model is available, the resolution obtained from the FSC between the model and experimental maps (*d*_FSC_model_; see Appendix *A* [App appa] for definitions) may provide another estimate for the resolution limit to which the data contain useful signal. The values of *d*_FSC_model_ generally match the values estimated from the recalculation of half-map correlations, *d*_FSC_, quite well (Fig. 9 ▸ *c*), although the values of *d*_FSC_model_ may be lower or higher than those of *d*_FSC_ depending on the FSC cutoff used. Note that the best correlation CC(*d*_FSC_, *d*_FSC_model_) is achieved for *d*_FSC_model_ calculated at FSC = 0.143. We note that this resolution metric is rather insensitive to the masking of the map (Fig. 9 ▸ *f*).

The second method (*d*_model_; ‘*Resolution estimate using atomic model*’ in §[Sec sec2.1.2]2.1.2) also uses the atomic model to estimate resolution, but unlike the previous method it does not use thresholds. Overall, *d*_model_ correlates with the reported resolution *d*_FSC_ (Fig. 9 ▸ *d*), although the *d*_model_ values are systematically larger, probably owing to accounting for atomic displacements or other disorder. A closer look at selected examples with the largest differences between these two values indicates that the appearance of the map is typically more in line with the estimated resolution *d*_model_ rather than with the reported *d*_FSC_ (see §[Sec sec3.6.4]3.6.4). It is possible that in some cases *d*_FSC_ may be reported not for the deposited map but for a map that has been manipulated in some way, for example masked; inversely, a masked map might be deposited while *d*_FSC_ is reported for the original map.

#### 3.6.3. Resolution estimates from map perturbation   

To investigate the question of resolution further, we explored removing high-resolution shells of Fourier coefficients and noting the resolution cutoff that we call *d*_99_ at which the map begins to change. Overall, these values correlate reasonably well with *d*_FSC_ (Fig. 9 ▸ *e*). However, for a number of structures *d*_99_ deviates from *d*_FSC_ rather substantially. Deviations with *d*_99_ > *d*_FSC_ indicate that the Fourier coefficients in the resolution range (*d*_FSC_, *d*_99_), though being accurate enough, are too weak to contribute significantly to the map. Deviations with *d*_99_ < *d*_FSC_ indicate the presence of Fourier coefficients of a resolution higher than *d*_FSC_ that significantly contribute to the map. Also, we note that map sharpening can affect *d*_99_ (Fig. 9 ▸ *g*) but it is rather insensitive to masking (Fig. 9 ▸ *h*).

#### 3.6.4. Analysis of selected examples with a large discrepancy between *d*_FSC_, *d*_99_ and *d*_model_   

Several examples below illustrate the utility and limitations of the resolution-estimation methods described in this manuscript (Table 1 ▸). We show that the differences between the various measures of resolution may originate from: (i) particular properties of the model and/or the data (map), (ii) annotation or some other procedural errors and (iii) limitations of the resolution metrics used.

**Figure 10:**
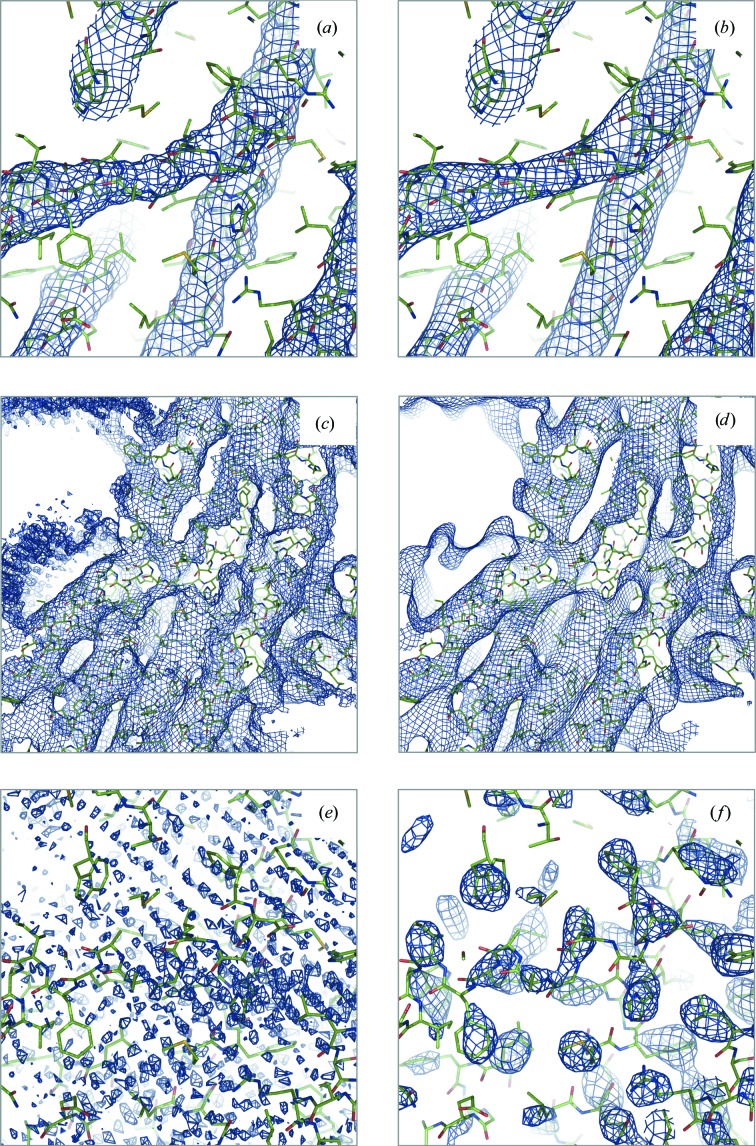
Maps for PDB entry 5UAR calculated by consecutive execution of the following steps: Fourier transform the original experimental map (EMDB code 8461), select a subset of Fourier coefficients of specified resolution range and finally calculate the new map using selected coefficients. Resolution ranges in Å: (*a*, *c*) 1.9–∞, (*b*, *d*) 6.7–∞, (*e*) 1.9–3.3, (*f*) 3.3–6.7. Pairs of maps (*a*, *b*) and (*c*, *d*) are the same maps shown at different contouring thresholds: high and low, respectively.

*Cystic fibrosis transmembrane conductance regulator (CFTR)*. The reported resolution for CFTR (Zhang & Chen, 2016 ▸; PDB entry 5UAR; EMDB map code 8461) is *d*_FSC_ = 3.7 Å. Visual inspection of the map suggests a significantly lower resolution (Fig. 10 ▸), which agrees with the model-based estimate of resolution *d*_model_ = 6.7 Å. At the same time *d*_99_ = 1.9 Å suggests that Fourier coefficients well beyond *d*_FSC_ are significant enough to affect the appearance of the map. The value of *d*_FSC_model_ calculated at FSC = 0 ranges between 3.3 and 3.6 Å (depending on whether sharpening or masking were used), suggesting that there is at least some correlation between model-derived and experimental maps up to this resolution. The original publication (Zhang & Chen, 2016 ▸) reports a local resolution varying between 2.6 and 6.0 Å.

**Figure 11:**
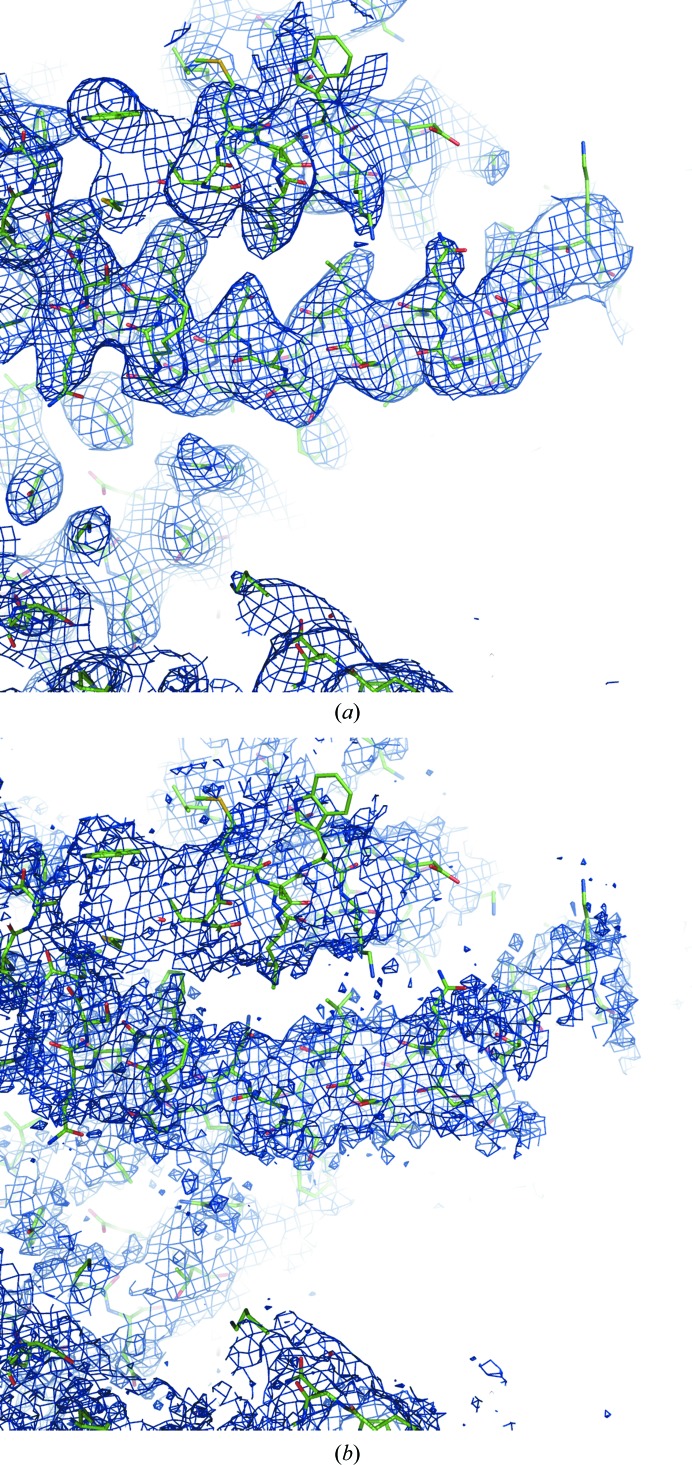
Sharpened maps for PDB entry 5UAR calculated similarly to as in Fig. 10 ▸ using data in the resolution ranges (*a*) 3.3–∞ Å (*B* = −240 Å^2^) and (*b*) 1.9–∞ Å (*B* = −20 Å^2^).

To investigate why these three resolution estimates report rather different values, we Fourier transformed the original map and then calculated four maps using subsets of the full set of map coefficients in the resolution ranges 1.9–∞, 6.7–∞, 1.9–3.3 and 3.3–6.7 Å (Fig. 10 ▸). Maps calculated using high-resolution cutoffs of 1.9 Å (or 3.3 Å, not shown) and 6.7 Å appear similar visually (Figs. 10 ▸ *a* and 10 ▸ *b*) except that the 6.7 Å resolution map is smoother and less noisy (Figs. 10 ▸ *a*, 10 ▸ *b*, 10 ▸ *c* and 10 ▸ *d*). A map calculated using Fourier coefficients in the 1.9–3.3 Å resolution range shows what appears to be artifacts or systematic noise throughout the box, which does not match features in the model (Fig. 10 ▸ *e*). This explains the value of *d*_99_ (1.9 Å): omitting this resolution range changes the map by eliminating (at least partially) this noise. This suggests that it may be reasonable to eliminate Fourier coefficients at this resolution to improve map quality before its interpretation. In contrast, a map calculated using the 3.3–6.7 Å resolution range (Fig. 10 ▸ *f*) shows many density features located essentially in the molecular region, with a majority of them, but not all, corresponding to the side chains of the deposited model. We note that these higher resolution features are not observed in the original map (even when contouring at very low cutoff values), being dominated by low-resolution data. This is confirmed by *d*_99_ = 6.5 Å calculated using the soft mask around the model (see Appendix *A* [App appa] for definition). Applying sharpening to the 3.3–∞ Å resolution map (sharpening *B* = −240 Å^2^) significantly improves it (Fig. 11 ▸ *a*), while any sharpening applied to the 1.9–∞ Å map makes the map deteriorate (Fig. 11 ▸ *b*; *B* = −20 Å^2^).

**Figure 12:**
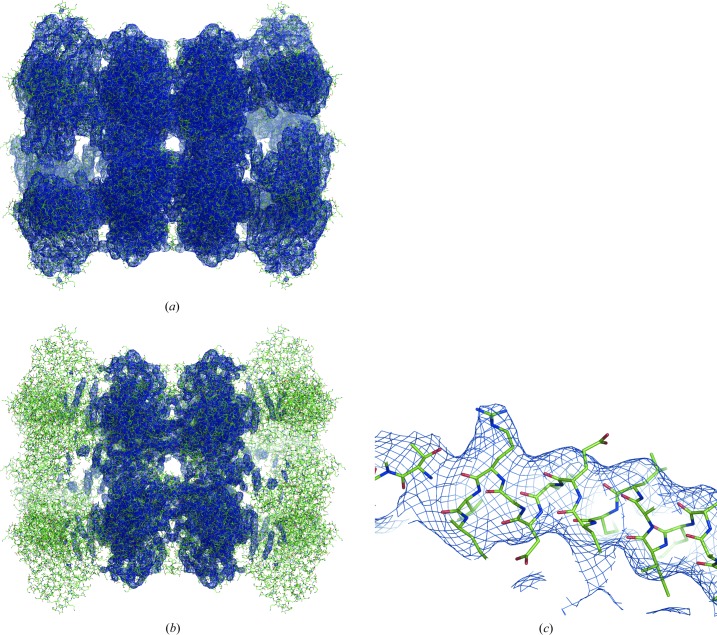
Maps for PDB entry 5LDF. (*a*) and (*b*) are shown with a low and high contouring threshold, respectively. (*c*) Fragment of a well resolved chain from a relatively high-resolution region, showing some side chains typical for resolutions of 4–4.5 Å (chain *B*, residues 435–460).

*Maltose-binding protein genetically fused to dodecameric glutamine synthet­ase*. In this example (Coscia *et al.*, 2016 ▸; PDB entry 5LDF; EMDB map code 4039), the map shows details specific for a resolution higher than the reported *d*_FSC_ = 6.2 Å. For example, a large number of side chains can be well distinguished (Fig. 12 ▸). Indeed, both suggested metrics give higher values: *d*_model_ = 4.0 Å, *d*_99_ = 4.4 Å. This means that for this structure Fourier coefficients of a resolution higher than *d*_FSC_ = 6.2 Å cannot be neglected. Indeed, the relevant article mentions that the resolution of the final reconstruction was 4.2 Å, in agreement with our calculations, and the local resolution varies between 10 and 3 Å, with the best-resolved regions being in the middle of the molecule (Fig. 12 ▸ *b*).

**Figure 13:**
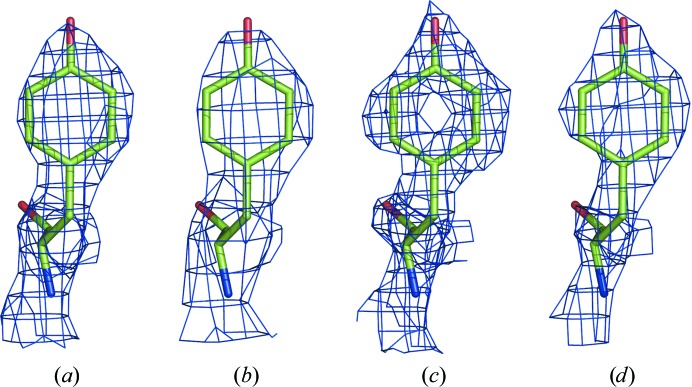
Maps for PDB entry 5K12 in the resolution ranges (*a*) 1.8–∞ Å, (*b*) 3–∞ Å, (*c*) 1.8–∞ Å sharpened with *B* = −35 Å^2^ and (*d*) 2.3–∞ Å sharpened with *B* = −38 Å^2^. Residue 382 in chain *A* is shown.

*Glutamate dehydrogenase*. For this example (Merk *et al.*, 2016 ▸; PDB entry 5K12; EMDB map code 8194), *d*_FSC_ = 1.8 Å and *d*_99_ = 1.9 Å, while *d*_model_ = 3.0 Å. This shows that even when Fourier coefficients are present up to a resolution of 1.8 Å and accurately defined, their contribution is relatively weak in comparison with other coefficients and the map appears more consistent with 3.0 Å resolution. Indeed, maps calculated using Fourier map coefficients in the ranges 1.8–∞ and 3–∞ Å appear essentially the same (Figs. 13 ▸ *a* and 13 ▸ *b*). Furthermore, the overall (CC_box_) and peak (CC_peak_) (Urzhumtsev *et al.*, 2014 ▸) correlations between these two maps are 0.96 and 0.86, respectively. For the model-calculated maps at 1.8 and 3 Å resolution these correlations are 0.88 and 0.40, respectively. This indicates that eliminating the 1.8–3 Å resolution range from the map coefficients has little effect on the original map. The resolution *d*_FSC_model_ obtained at FSC = 0, 0.143 and 0.5 is 1.8, 2.3 and 3 Å, respectively, which confirms that there is some signal in this range but it is just weak. A sharpened map at 1.8–∞ Å (Fig. 13 ▸ *c*) shows details expected at resolutions around 2 Å, and truncating the data to 2.3–∞ Å does change the map visibly (Fig. 13 ▸ *d*). We note that not all regions of the volume behave similarly to as in this example (Fig. 13 ▸) because the resolution varies across the volume, with 1.8 Å resolution for the best parts. This explains the small difference in the correlations calculated between 1.8 and 3.0 Å filtered maps.

**Figure 14:**
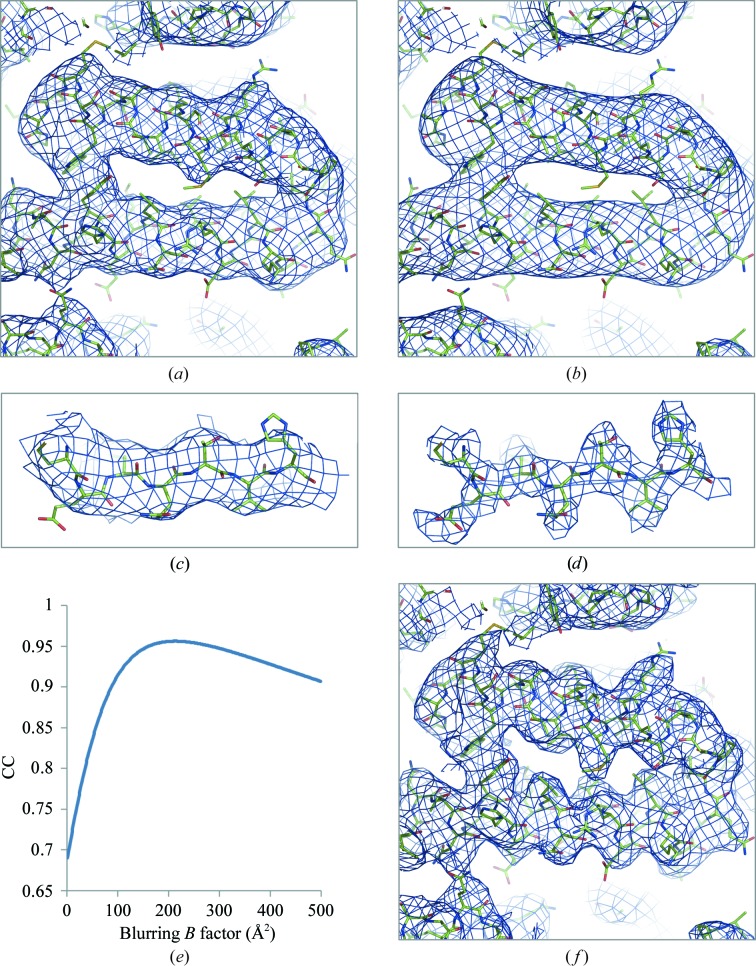
Maps for PDB entry 5K7L: (*a*) original and (*b*) calculated using Fourier map coefficients in the 7.4–∞ Å resolution range. (*c*) The original map and (*d*) the map calculated using 3.6–7.4 Å resolution data are shown for residues 568–574. (*e*) Correlation between 7.4 Å resolution and overall *B*-­factor-blurred 3.8 Å resolution model-calculated maps as a function of blurring *B*-factor. (*f*) Sharpened original map.

*Voltage-gated K^+^ channel Eag1*. This is a case (PDB entry 5K7L; EMDB map code 8215; Whicher & MacKinnon, 2016 ▸) in which the resolutions reported in the map (*d*_FSC_) and estimated using the model (*d*_model_) match at a value of 3.8 Å, while *d*_99_ = 7.4 Å. Performing similar calculations as those carried out for CFTR above, we find that the original map (Fig. 14 ▸ *a*) and the map calculated using a resolution range of 7.4–∞ Å (Fig. 14 ▸ *b*) appear to be essentially the same except for small hints of side chains in the higher resolution map. Inspecting the original map at lower contour levels does not reveal any more information for the side chains. Calculating a map using the 3.8–7.4 Å resolution range results in a map that is expectedly noisy overall but also clearly shows side chains for many residues (Fig. 14 ▸ *d*) when compared with the original map (Fig. 14 ▸ *c*). The discrepancy between *d*_FSC_ and *d*_99_ is likely to be because the map is dominated by the low-resolution data and omitting high-resolution terms does not change the map significantly enough for the *d*_99_ metric. Calculating *d*_model_ includes the optimization of an overall *B* factor (Appendix *B* [App appb]), which was found in this case to be 260 Å^2^. This rather large overall *B* factor may provide an additional explanation of the difference between estimated resolutions. Indeed, it is known that image blurring by application of a *B* factor acts similarly to lowering the resolution cutoff. The following example illustrates this. Using the 5K7L model, we reset all *B* factors to 0 and calculated two maps at 3.8 and 7.4 Å resolution. We then sampled *B* factors in the range 0–500 Å^2^ and applied each trial *B* factor as an overall blurring *B* factor to the 3.8 Å resolution map. Fig. 14 ▸(*e*) shows the correlation between the 7.4 Å resolution map and the overall *B* factor-blurred 3.8 Å resolution map as a function of the blurring *B* factor. The maximum CC is at 213 Å^2^, which is in the same range as the overall *B* factor obtained during the *d*_model_ calculation. Map sharpening is expected to reduce blurring owing to an overall *B* factor. Indeed, applying an automated sharpening procedure (*phenix.auto_sharpen*; Terwilliger *et al.*, 2018 ▸) results in a map with significantly enhanced details (Fig. 14 ▸ *f*) that are expected at 3–4 Å resolution. We also note that while the sharpened map shows more detail (as expected in this case; compare Figs. 14 ▸ *a* and 14 ▸ *f*), all three model–map correlations (CC_mask_, CC_volume_ and CC_peaks_) are lower for the sharpened map (0.749, 0.745 and 0.495, respectively) compared with the original map (0.810, 0.803 and 0.559, respectively).

#### 3.6.5. Recommendations for use of the metrics presented   

The examples above illustrate the different metrics discussed in this article. These metrics are summarized in Tables 2 ▸ and 3 ▸. Below, we provide practical suggestions for the use of these metrics.

Once a three-dimensional reconstruction is available, *d*_99_ can be calculated and compared with *d*_FSC_. If *d*_99_ is significantly smaller than *d*_FSC_ then this indicates the presence of Fourier coefficients in the resolution shell *d*_99_ ≤ *d* < *d*_FSC_ that can be considered as less reliable according to *d*_FSC_. They may need to be filtered out or used with caution. It may also be prudent to verify the value of *d*_FSC_ obtained from the FSC curve calculated using half-maps.

If *d*_99_ is significantly larger than *d*_FSC_ then this indicates relative weakness of the data within the resolution limits *d*_FSC_ ≤ *d* < *d*_99_. Since these data are considered as reliable according to the chosen *d*_FSC_, this suggests that the map in question may benefit from an appropriate attenuation, *i.e.* sharpening or filtering.

Once an atomic model is available, *d*_model_ can be calculated and compared with *d*_FSC_ and *d*_99_. A significant difference between these values, as shown in the examples above, may be indicative of structural and/or map peculiarities, for example unusual atomic displacement parameters or a strongly non-uniform resolution across the map volume.

It may happen that the original map with no masking or sharpening applied may not visually convey the actual information content. For example, no side chains may be visible in the original map, while they may be visible in a sharpened or filtered map, as the examples above show. This situation can be detected by *d*_FSC_model_, which is generally expected to be greater than *d*_FSC_. Weak but accurate map details interpreted by a correct model will result in high FSC values for all resolutions up to *d*_FSC_, *i.e.* making *d*_FSC_model_ ≃ *d*_FSC_. In situations where *d*_FSC_model_ < *d*_FSC_ it may be necessary to re-evaluate the *d*_FSC_ value. Assuming that the atomic model correctly fits the map overall, *d*_FSC_model_ provides an objective measure of the resolution limit up to which there is at least some signal arising from the model that correlates with the map. Also, *d*_FSC_model_ is independent of map sharpening or blurring.

After a model has been built, one can calculate real-space correlation coefficients, as discussed above. For a correct and complete model, all three values, CC_mask_, CC_volume_ and CC_peaks_, are expected to be high, for example greater than 0.7–0.8. Low values of CC_mask_ or CC_volume_ indicate disagreement between the model and the experimental maps (see below), in turn suggesting revision of the atomic model. If the model is deemed to be correct, the steps and procedures used to obtain the experimental map should be reviewed. CC_mask_ and CC_volume_ reflect the model-to-map fit in two related but still different regions. CC_mask_ compares model-calculated and experimental density around atomic centers, with atomic centers being inside the regions used to calculate CC_mask_. CC_volume_ compares model-calculated and experimental density inside the molecular envelope but not necessarily around atomic centers, as peaks in low-resolution Fourier images do not necessarily coincide with atomic positions. When CC_mask_ is high but CC_volume_ is low, the map may have been over-sharpened overall or locally.

The values of CC_mask_ and CC_volume_ may be surprisingly low if the model obtained from analysis of sharpened maps is then compared with the original map that contains accurate but weak high-resolution features; this inspired the work of Urzhumtsev *et al.* (2014 ▸).

When both CC_mask_ and CC_volume_ are acceptably high, a low value of CC_peaks_ indicates model incompleteness (*i.e.* the presence of peaks in the experimental map that are not explained in terms of the atomic model) or artifacts in the region of the experimental map outside the model.

There are a multitude of methods and software to sharpen or blur maps. Additionally, particular procedures may require different map manipulations. For example, automated model building may benefit from map blurring at some stages to facilitate secondary-structure identification and placement in the map. Further model building and refinement may require map sharpening in order to locate, place and refine other model details, such as side chains. Estimating map resolution using FSC-based methods may require map masking, and there are several methods and software packages that perform this. While FSC-based measures are indeed insensitive to scaling, they are sensitive to masking. With the current state of the art, it is essentially impossible to track and reproduce all of these possible manipulations that have been applied to a map. With this in mind, we believe that the original maps should be used to obtain statistics. Additionally, a set of statistics can also be reported for whatever manipulated map was used in obtaining the final deposited atomic model.

### 3.7. Model bias   

Depending on the method used to determine an atomic model, bias may be an issue. In crystallographic structure determination, a model almost always feeds back into the structure-determination process by providing valuable phase information. Multiple methods have been developed to identify and combat model bias (for example, Bhat & Cohen, 1984 ▸; Read, 1986 ▸; Brünger, 1992 ▸; Hodel *et al.*, 1992 ▸). Therefore, while model bias is a serious permanent and recognized problem in crystallography, there are ways to mitigate it much of the time, although these methods are increasingly challenged as the data resolution worsens.

In cryo-EM the situation is radically different. At present, unless specific methods are used (Jakobi *et al.*, 2017 ▸), there is no point to the process where an atomic model is fed back into the structure-determination process. Direct observation of a real image in the microscope makes it possible to obtain the phase information experimentally. Therefore, the map that is used to build and refine a model is static, being derived without ever ‘seeing’ an atomic model. Thus, the problem of model bias is nonexistent in this sense. However, when combining two-dimensional projections into a three-dimensional image, a previously determined model may be used as an initial reference structure; this may result in a map showing features that are present in the reference structure and not in the experimental cryo-EM images. This aspect of model bias has been discussed, for example, by van Heel (2013 ▸), Subramaniam (2013 ▸), Henderson (2013 ▸) and Mao *et al.* (2013 ▸), and is beyond the scope of the current work.

### 3.8. Overfitting and multiple interpretation   

Both the model-bias and overfitting problems in cryo-EM have been discussed by Rosenthal & Rubinstein (2015 ▸). Overfitting may result in a model that explains the data well but is in fact incorrect, either in whole or in part. A classic example is using a model with more parameters than data. In the crystallographic process, since model bias is inherent and the amount of observed data is often limited, both factors contribute to potential overfitting. Introduction of cross-validation using a free *R* factor (Brünger, 1992 ▸) has provided tools to identify and reduce the overfitting. However, the problem becomes increasingly challenging with low-resolution data.

In cryo-EM the problem of overfitting occurs when atomic model details are not confirmed by the experimental data (map reconstruction) or simply match noise in the map. It is worth thinking about the effective data content for crystallo­graphic data and a cryo-EM map at the same resolution. In crystallographic cases, if we consider a complex plane representation of an observation in Fourier space, models with any phase are all equally consistent with the data, where there is often only amplitude information. In contrast, the cryo-EM case has both amplitude and phase information from the experiment, and the possible set of models is significantly more constrained (there is about twice as much information in the cryo-EM map if experimental phase information is not present in the crystallo­graphic case). In either case, however, there is still the possibility of constructing models that have a good fit to the data, especially with low-resolution data, but are incorrect, at least in part.

Although a free *R* factor can be calculated for a cryo-EM model, there are inherent challenges in this approach. Conversion of the map to a reciprocal-space representation is possible, but the *R*-factor value depends on the choice of the box around the macromolecule, masking around the molecule, use of the entire box of Fourier coefficients *versus* a sphere with the radius based on the resolution (if crystallographic tools are used, for example), and other factors including the correlations between neighboring voxels in the map arising from the three-dimensional reconstruction procedure. The practice of calculating an FSC between one half-map and a map calculated from a model refined against another half-map is routinely used to assess whether the model is fitting noise (for example, DiMaio *et al.*, 2009 ▸; Brown *et al.*, 2015 ▸; Chang *et al.*, 2015 ▸; Nguyen *et al.*, 2016 ▸). This falls short of detecting overfitting in the case of an incorrect model because the model may have the wrong atoms placed in a particular region of correct density. Also to address the overfitting problem, Chen *et al.* (2013 ▸) suggested comparing the FSC obtained using the original data with the FSC obtained using modified data with noise introduced into the highest resolution Fourier coefficients.

**Figure 15:**
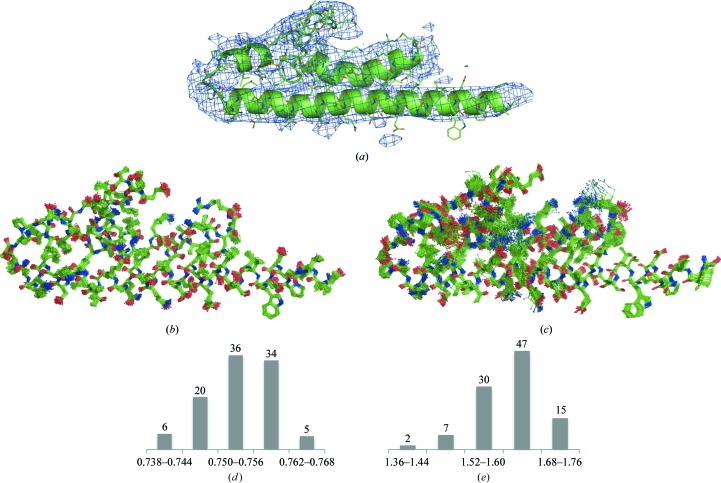
Illustration of multiple interpretation. (*a*) PDB entry 3J0R and the corresponding map (EMDB code 5352). (*b*) Ensemble of 100 perturbed models obtained using MD; all models in the ensemble deviate from the starting model by 0.5 Å. (*c*) Real-space refined models obtained from (*b*) using *phenix.real_space_refine*. (*d*) Distribution of model–map correlation for refined models. (*e*) Distribution of r.m.s. deviations between starting and refined models.

Low resolution provides room not only for data overfitting but also for multiple possible interpretations of the data, with models that fit the data equally well and that are equally meaningful physically and chemically (Pintilie *et al.*, 2016 ▸). In turn, differences between multiple models (Rice *et al.*, 1998 ▸) could be used to detect regions that are misfitted or where the map quality is poor. One approach to assessing the uniqueness of the map interpretation is to explicitly create multiple models that are all consistent with the data (Terwilliger *et al.*, 2007 ▸; Volkmann, 2009 ▸). To assess multiple interpretations of maps, we made the tools described in Afonine *et al.* (2015 ▸) available as a utility called *phenix.mia* (where MIA stands for multiple interpretation assessment). Essentially, this utility performs the steps described in §3.7 of Afonine *et al.* (2015 ▸) in an automated way to generate an ensemble of refined models. A subset of models is then selected such that all selected models fit the map equally well. Finally, deviations between the same atoms of selected models are analyzed. A similar approach that incorporates automated model rebuilding has also recently been described (Herzik *et al.*, 2017 ▸). We stress that making multiple models reports on precision (uncertainty) and not accuracy. It is also convoluted with the limitations of refinement and sampling (Terwilliger *et al.*, 2007 ▸). For an illustration, we took the 3J0R model (EMDB map 5352) that has a modest resolution of 7.7 Å (Fig. 15 ▸ *a*). Using *phenix.mia*, we generated an ensemble of 100 slightly perturbed models (shown in Fig. 15*b*) by running independent MD simulations, each starting with a different random seed, until the r.m.s. difference between the starting and simulated models was 0.5 Å. The procedure then subjected each model to real-space refinement using *phenix.real_space_refine* (Afonine, Headd *et al.*, 2013 ▸; Afonine *et al.*, 2018 ▸) until convergence. This resulted in 100 refined models, as shown in Fig. 15 ▸(*c*). While these refined models are different, having r.m.s. deviations from the starting model ranging between 1.4 and 1.8 Å (Fig. 15 ▸ *e*), none of them has geometric violations and they all have a similar fit to the map (Fig. 15 ▸ *d*). We can therefore draw the conclusion that the uncertainty in atomic coordinates (positional uncertainty, not in individual *x*, *y* and *z*) after interpretation of this map is on the order of at least 1.4–1.8 Å.

### 3.9. Re-refinement of selected models   

In this work, we identified a number of issues present in currently available cryo-EM depositions. Some of them would require a considerable amount of manual intervention to address. These include missing map box information (known as unit-cell parameters in the crystallographic context), a lack of or invalid MTRIX or BIOMT matrices, and incorrect secondary-structure annotations. Other issues, such as model-geometry violations, poor model-to-map fit or unrealistic ADPs or/and occupancy factors, can be addressed in an automated or semi-automated way using current tools. To illustrate the point, we selected a number of models among those with the highest number of geometry outliers and performed a round of real-space refinement using *phenix.real_space_refine*. Table 4 ▸ shows that in all cases the number of geometric violations was significantly reduced, and in many cases was reduced to zero. Moreover, the model-to-map fit quantified here by CC_mask_ was improved in many cases as well. In some cases, however, CC_mask_ remained unchanged or decreased slightly. This suggests that the original model, before refinement, was overfitting the data, *i.e.* better fitting the data at the expense of distortions in model geometry. Therefore, we consider the decreased correlation in such cases to still be an improvement. We also note that not all geometry outliers were removed by refinement. One of reasons is that gradient-driven refinement is a local optimization process with a limited convergence radius. Given the number and severity of geometry violations in some of the cases, it is expected that some of them are not fixed by simple refinement but would rather require local model rebuilding first.

## 4. Conclusions   

Crystallography and cryo-EM are similar in the sense that both yield an experimental three-dimensional map to be interpreted in terms of a three-dimensional atomic model. In crystallography the experimental data are diffraction intensities, and in cryo-EM the data are three-dimensional objects reconstructed from two-dimensional projections acquired from the microscope. Once an initial map (Fourier image of electron or nuclear density distribution in crystallography) or three-dimensional reconstruction (image of electrostatic potential in cryo-EM) is obtained, the next steps leading to the final refined atomic model are very similar. Integral to these steps is validation of the data, the atomic model and the fit of the atomic model to the data. However, since the types of experimental data are different, the two methods require different validation approaches.

The goal of this work was threefold. Firstly, we wanted to identify what is lacking in the arsenal of validation methods and to begin filling the gaps by developing new methods. Secondly, we wanted to exercise existing or newly added tools by applying them to all available data in order to assess their utility and robustness. Finally, we wanted to obtain an overall assessment of the data, model and model-to-data fit quality of cryo-EM depositions currently available in the PDB and the EMDB. Similar work has been performed for crystallographic entries in the past (see, for example, Afonine *et al.*, 2010 ▸), but not yet for cryo-EM; a subset of cryo-EM maps has recently been analyzed by Joseph *et al.* (2017 ▸). The scope of this validation is global in a sense that we calculated and analyzed overall statistics for the model and the data.

As a result of our analysis, we advocate for a formal and uniform procedure for validation of atomic models obtained by cryo-EM, as is nowadays available in macromolecular crystallography (Gore *et al.*, 2012 ▸), including a cryo-EM-specific validation report, which could be an extension of those currently generated by the wwPDB OneDep system (Young *et al.*, 2017 ▸). The lack of such a procedure may result in incorrect interpretations and misuse of deposited atomic models. As in crystallography, the deposited information should be sufficient to reproduce the validation tests. In particular, this requires the presence of half-maps and the mask used for FSC and model–map correlation calculations. It would be preferable to establish a universal procedure for the mask calculation. Also, when reporting values of some metrics, these should be clearly defined and, if possible, commonly accepted by the community and used in the same way for reproducibility and compatibility between different software packages. We envisage a Summary Table similar to the widely accepted crystallographic ‘Table 1’, which would include information about the highest resolution shell of a Fourier space, including FSC for half-maps, FSC map–model and relative strength of amplitudes in comparison to other resolution shells. Some other metrics, for example those discussed in Tickle (2012 ▸), can also be included.

There is an opportunity to address some of the current limitations in the validation of cryo-EM maps and the models derived from them before the database grows significantly in size. Improvements in the deposition process would minimize some of the inconsistences in models and maps that we have observed. Cryo-EM reconstructions have reached a resolution that warrants rigorous checks on coordinates, atomic displace­ment parameters and atomic occupancies. These need to be combined with well established measures of stereochemistry, and new cryo-EM-specific methods that compare the model and the map, for example *EM-Ringer*. It is essential that community-agreed standards are developed for the data items to be deposited by researchers. Our analysis shows that for validation the mask used to calculate *d*_FSC_ should be deposited along with the map and the two half-maps. The question of resolution will no doubt remain a subject of some debate, but providing the appropriate information at the time of structure deposition will greatly enhance the ability of other researchers to assess resolution. Ultimately, clearly defined validation procedures will help to highlight even further the increasing contribution of high-resolution cryo-EM to the field of structural biology.

## Figures and Tables

**Figure 8:**
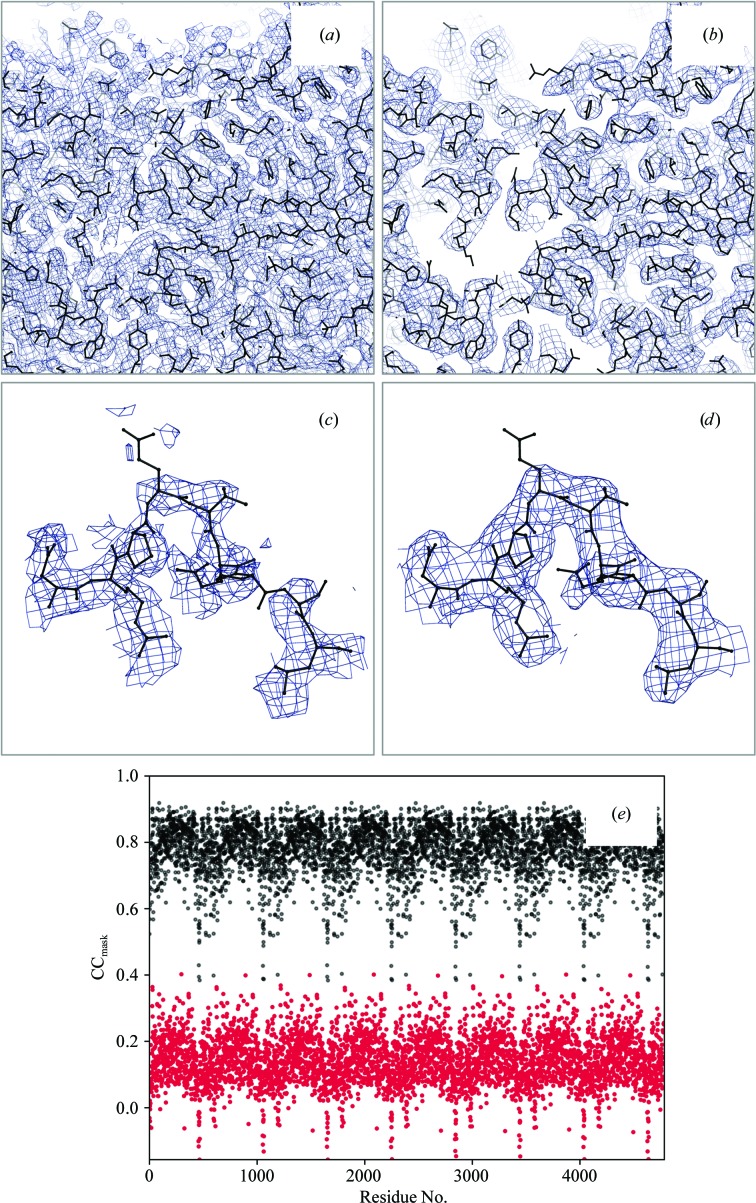
(*a*, *c*) An apparently over-sharpened map (PDB and EMDB codes 5NV3 and 3699, respectively; resolution 3.39 Å). Applying *phenix.auto_sharpen* improves the map by blurring it. (*b*, *d*) Subsequent refinement against the blurred map improves the model-to-map fit, as shown by CC_mask_ reported per residue (*e*) (black dots).

**Figure 16:**
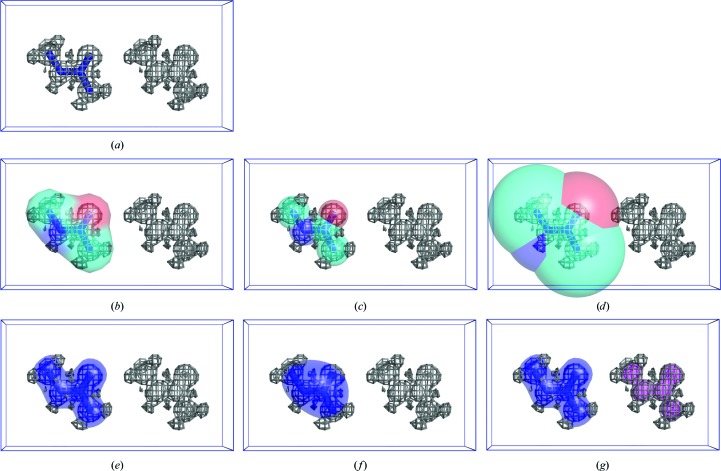
Illustration of different subsets of the grid nodes used to calculate the correlation coefficients between model and target maps. (*a*) Atomic model (blue sticks) superposed with partially interpreted target map (gray); the correlation coefficient CC_box_ between the target and model map is calculated over the whole cell. (*b*) Molecular mask calculated by Jiang & Brünger (1994 ▸), CC_mask_. (*c*, *d*) Mask derived from atomic images at higher and lower resolutions, CC_image_. (*e*, *f*) Peaks within the given volume in higher and lower resolution model maps CC_volume_. (*g*) Mask derived from the peaks of the model (blue) and target (magenta) maps, CC_peaks_; the total mask is the union of the blue and magenta masks.

**Figure 17:**
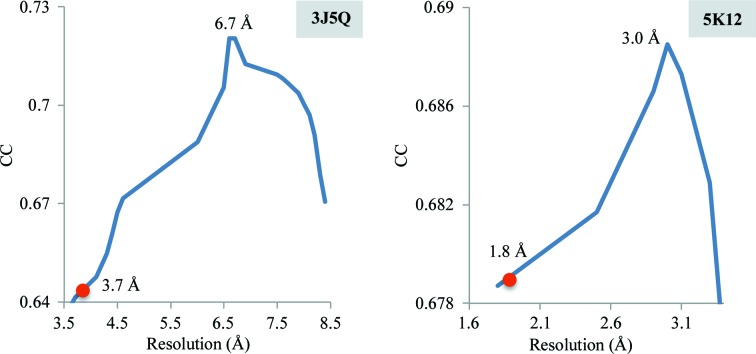
Correlation coefficient between an experimental map and maps generated from the model at different resolutions, shown for selected PDB entries. The red circle on each curve indicates the reported resolution, *d*_FSC_, and the number on the top of the peak indicates the estimated resolution.

**Figure 18:**
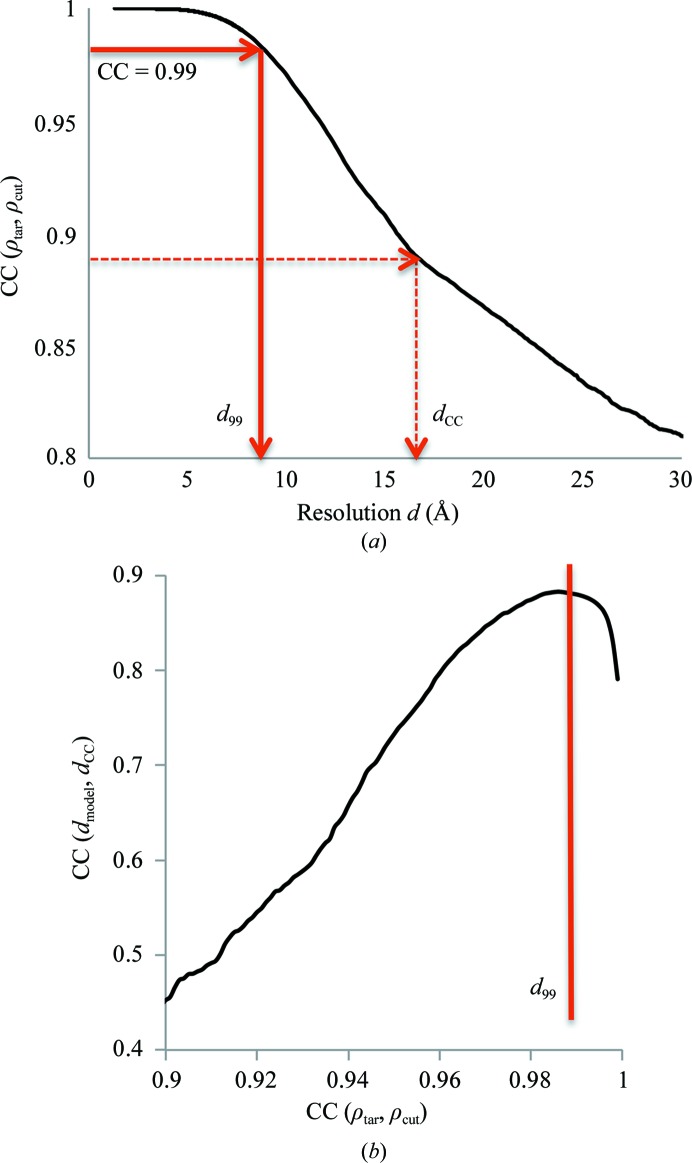
(*a*) Correlation coefficient [equation (4), Appendix *C* [App appc]] between the original map and a high-resolution truncated map shown as a function of the resolution value used for truncation for PDB entry 3J27. *d*_99_ corresponds to CC = 0.99. (*b*) Correlation coefficient between *d*_model_ and trial resolution cutoffs *d*_CC_, calculated using all selected data sets, shown as function of CC(ρ_tar_, ρ_cut_). See Appendix *C* [App appc] for details.

**Figure 19:**
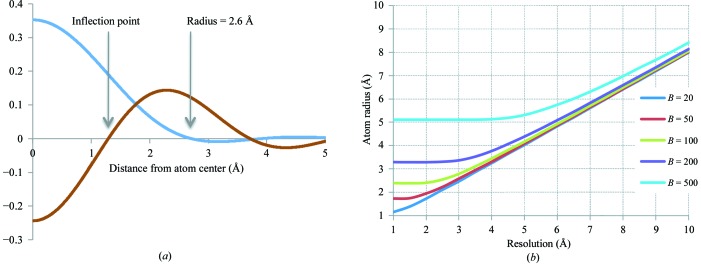
(*a*) 3 Å resolution Fourier image of a C atom with *B* factor 50 Å^2^ (blue) and its second derivative (brown); the image is spherically symmetric and is represented by a one-dimensional radial distribution. The atom radius is defined as twice the distance from the center of the atom to the first inflection point of this curve. (*b*) Radius as determined in (*a*) for the C atom as a function of resolution, shown for several *B*-factor values.

**Figure 20:**
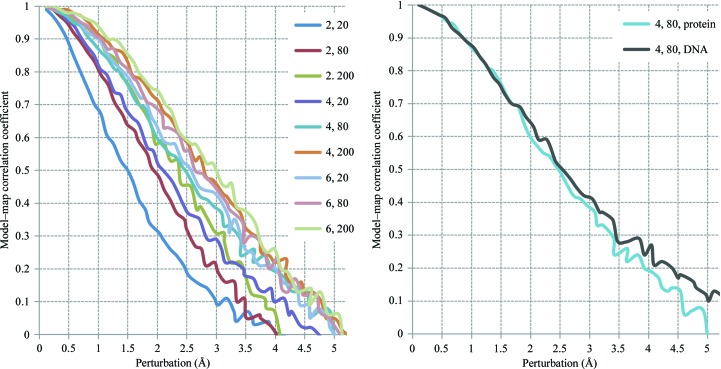
Model–map correlation coefficient calculated between a target map and the map from a perturbed model shown as function of perturbation at different resolutions (2, 4 and 6 Å) and different overall ADPs (20, 80 and 200 Å^2^). Left, a protein model. Right, copy of a curve for the protein model taken from the left picture (light blue) and the corresponding curve obtained at the same resolution and ADP for an RNA molecule; this illustrates the low dependence of the results on the choice of molecule.

**Table 1:** Resolution metrics for selected examples

		Maps		
		Not masked	Masked	
Map, model and metrics	Reported *d*_FSC_	Original	Original	Sharpened
PDB entry 5UAR †, EMDB code 8461				
*d*_99_		1.9	6.5	4.7
*d*_model_ (*B*, Å^2^)	3.7	6.7 (−60)	6.7 (−10)	6.6 (−90)
*d*_model(*B*=0)_		6.6	6.7	6.5
*d*_FSC_model_		3.6	3.3	3.4
PDB entry 5LDF ‡, EMDB code 4039				
*d*_99_		4.4	4.9	4.1
*d*_model_ (*B*, Å^2^)	6.2	4.0 (220)	4.1 (220)	4.1 (−10)
*d*_model(*B*=0)_		7.5	7.4	4.2
*d*_FSC_model_		3.6	3.5	3.5
PDB entry 5K12 §, EMDB code 8194				
*d*_99_		1.9	2.5	2.8
*d*_model_ (*B*, Å^2^)	1.8	3.0 (20)	3.0 (20)	3.0 (10)
*d*_model(*B*=0)_		3.4	3.4	3.3
*d*_FSC_model_		1.8	1.8	2.0
PDB entry 5K7L ¶, EMDB code 8215				
*d*_99_		7.4	6.9	3.9
*d*_model_ (*B*, Å^2^)	3.8	3.6 (260)	3.6 (300)	3.8 (40)
*d*_model(*B*=0)_		8.3	8.6	4.0
*d*_FSC_model_		3.5	3.2	3.4

† The original map for PDB entry 5UAR contains high-resolution features (likely to be noise) outside the model. These features can be removed by masking (compare *d*_99_ for the masked and unmasked maps). The unsharpened map does not show higher resolution details (see *d*_model_). The model reproduces all details up to *d*_FSC_ (compare *d*_FSC_ and *d*_FSC_model_). High-resolution filtering followed by sharpening may be required to build and confirm these details.

‡ The original map for PDB entry 5LDF contains details of a resolution higher than *d*_FSC_ (compare *d*_FSC_ and *d*_99_); the molecular region also contains these details (compare *d*_99_ for the masked and unmasked maps). The unsharpened map indeed looks like a map nearer 6 Å resolution (the difference between *d*_model_ calculated with underestimated *B* = 0 and overestimated *B* = 220 Å^2^). The model reproduces details up to a resolution slightly higher than 4 Å (see *d*_FSC_model_), which is confirmed by all metrics calculated for the sharpened map. It is possible that *d*_FSC_ is underestimated.

§ The original map for PDB entry 5K12 contains high-resolution details up to *d*_FSC_ (*d*_99_ for the unmasked map). Inside the molecular region neither the original nor the sharpened map show such details (*d*_99_ for masked maps) and the map itself looks like a 3 Å resolution map (see *d*_model_). At the same time, the model reproduces the data up to a resolution near *d*_FSC_ (*d*_FSC_model_). To visualize these details, the default sharpening is insufficient and omitting dominating lower resolution data may be needed.

¶ The original data for PDB entry 5K7L are weak at higher resolution and the original map shows limited detail (low *d*_99_ for unsharpened maps); these details do appear in the sharpened map (compare *d*_99_ and *d*_FSC_, also compare *d*_99_ for sharpened and unsharpened maps). Indeed, the original map in the molecular region is blurred by very large *B* [compare *d*_model_ and *d*_model(*B*=0)_]. The sharpened map looks like a map at *d*_FSC_ [compare *d*_FSC_ with *d*_model_ and *d*_model(*B*=0)_ for sharpened maps]. The model reproduces the map details well (compare *d*_FSC_model_ and *d*_FSC_).

**Table 2:** Summary of map resolution estimates

Metric	Objects used	Purpose	Values	Meaning, possible actions
*d*_FSC_	Half-maps	Highest resolution at which the experimental data are confident	The higher the better	Resolution determined using half-maps method
*d*_99_	Map	Resolution cutoff beyond which Fourier coefficients are negligibly small	*d*_99_ ≥ *d*_FSC_	Expected values
			*d*_99_ < *d*_FSC_	Verify *d*_FSC_; omit coefficients with *d*_99_ ≤ *d* < *d*_FSC_
			*d*_99_ >> *d*_FSC_	Sharpen the map
*d*_model_	Map and model	Resolution cutoff at which the model map is the most similar to the target map	*d*_model_ ≥ *d*_FSC_	Expected values
			*d*_model_ < *d*_FSC_	Verify *d*_FSC_; check ADP (too large?); validate map details
			*d*_model_ >> *d*_FSC_	Sharpen the map
			*d*_model_ << *d*_99_	Check ADP (too large?)
			*d*_model_ >> *d*_99_	Check ADP (too small?); check the model
*d*_FSC_model_	Map and model	Resolution cutoff up to which the model and map Fourier coefficients are similar	*d*_FSC_model_ ≥ *d*_FSC_	Expected values
			*d*_FSC_model_ < *d*_FSC_	Verify *d*_FSC_; omit coefficients with *d*_FSC_model_ ≤ *d* < *d*_FSC_
			*d*_FSC_model_ ≥ *d*_FSC_	Sharpen the map
			*d*_FSC_model_ >> *d*_model_	Omit coefficients with *d*_model_ ≤ *d* < *d*_FSC_model_
			*d*_FSC_model_ << *d*_model_	Sharpen the map

**Table 3:** Summary of map correlation coefficients used in this work

Metric	Region of the map used in calculation	Purpose
CC_box_	Whole map	Similarity of maps
CC_mask_	Jiang & Brünger (1994 ▸) mask with a fixed radius	Fit of the atomic centers
CC_volume_	Mask of points with the highest values in the model map	Fit of the molecular envelope defined by the model map
CC_peaks_	Mask of points with the highest values in the model and in the target maps	Fit of the strongest peaks in the model and target maps
CC_vr_mask_	Same as CC_mask_ but atomic radii are variable and function of resolution, atom type and ADP	Fit of the atomic images in the given map

**Table 4:** Re-refinement of selected models that have among the highest numbers of geometry outliers Columns show, from left to right: PDB and EMDB codes for the model and map, resolution as extracted from the EMDB and statistics calculated before and after refinement using *phenix.real_space_refine*. The statistics include the map correlation coefficient CC_mask_, r.m.s. deviations from ideal (library) values for covalent bonds and angles, Ramachandran plot and residue side-chain rotamer outliers, the percentage of C^β^ deviations and the *MolProbity* clashscore.

		Before/after refinement						
PDB, EMDB code	Resolution (Å)	CC_mask_	R.m.s.d., bonds (Å)	R.m.s.d., angles (°)	Ramachandran outliers (%)	Rotamer outliers (%)	C^β^ deviations (%)	Clashscore
3J9i, 5623	3.3	0.77/0.76	0.034/0.009	3.61/1.38	1.9/0.7	9.3/2.1	10.4/0	5.3/4.7
3J27, 5520	3.6	0.62/0.57	0.009/0.009	1.96/1.79	24.5/0.9	20.1/2.8	0.1/0.1	112.2/10.8
5J8V, 8073	4.9	0.67/0.69	0.024/0.008	2.67/1.40	7.3/1.0	28.7/5.2	1.6/0	71.2/1.9
5AKA, 2917	5.7	0.37/0.46	0.014/0.008	2.14/1.74	18.9/0.6	26.7/1.9	0.7/0	74.5/5.0
5SV9, 8313	5.9	0.78/0.70	0.041/0.009	4.00/1.52	5.9/0	20.0/2.0	16.3/0	42.1/7.8
3J5L, 5771	6.6	0.62/0.53	0.011/0.008	1.73/1.68	11.4/0.7	25.6/1.8	0.6/0.1	67.1/5.7
5HNW, 8058	6.6	0.68/0.71	0.020/0.007	1.95/1.31	11.6/0.1	13.2/0.8	0.7/0	82.1/8.6
4V5M, 1798	7.8	0.58/0.47	0.029/0.010	2.89/1.75	11.9/0.5	14.9/1.9	1.1/0	64.8/8.5
2J28, 1262	8.0	0.29/0.30	0.034/0.008	2.72/1.69	20.4/0.5	24.4/2.4	0.6/0.1	91.6/6.5
3iYF, 5140	8.0	0.72/0.67	0.043/0.008	6.48/1.57	13.7/0.3	40.9/2.0	55.1/0.4	80.6/6.7
4AAQ, 1998	8.0	0.54/0.73	0.023/0.011	2.52/1.53	0.2/0	10.0/2.1	11.7/0	11.8/15.3
4AAR, 1999	8.0	0.52/0.71	0.019/0.009	2.50/1.37	0.3/0	9.5/1.1	11.6/0	7.9/12.3
4V6T, 5386	8.3	0.53/0.42	0.016/0.008	1.81/1.55	11.5/0.2	22.9/2.0	0.2/0	58.5/8.1
4ABo, 2005	8.6	0.63/0.82	0.018/0.008	1.88/1.37	12.9/0.1	16.8/1.0	0.2/0	93.1/8.2
3iY4, 5109	11.7	0.67/0.70	0.031/0.006	3.95/1.14	6.0/0.5	9.4/0.6	9.5/0	80.9/7.8
4CKD, 2548	13.0	0.60/0.74	0.018/0.007	2.64/1.18	0.5/0.4	12.6/0.4	9.4/0	25.5/12.5
3iY7, 5112	14.0	0.77/0.76	0.025/0.007	3.09/1.32	6.0/0	8.4/1.6	13.0/0	76.0/10.9

## Footnotes

1 In the case of crystallography, this is performed using the same set of reflections as in the observed data set, which accounts for data completeness.

2 The PDB and ligand codes are written following the convention outlined in Moriarty (2015 ▸).

3 Reported at resolution *d*_FSC_ = 2.9 Å, the coefficients are CC_volume_ = 0.62 and CC_mask_ = 0.75; other examples are PDB entry 5K0U (*d*_FSC_ = 2.8 Å; CC_volume_ = 0.69, CC_mask_ = 0.84) and PDB entry 5AC9 (*d*_FSC_ = 3.2 Å; CC_volume_ = 0.73, CC_mask_ = 0.89).

## Appendix A Correlation coefficients and regions of their calculation

Correlation coefficients calculated with different subsets of grid nodes {**n**} answer different questions, may have different values and describe different aspects of model-to-map fit (or lack thereof).

Below, we define five types of real-space correlation coefficients, each differing in the choice of map regions (masks) that are used to calculate them. For most of these masks it is possible to adjust their parameters in ways that may result in higher or lower values of the corresponding correlation coefficients. Additionally, we describe how we calculate a map correlation coefficient in reciprocal (Fourier) space: Fourier shell correlation (FSC). While FSC itself is a well established metric, there are a number of nuances pertinent to its calculation that are important to state in order to make it reproducible.

### A1. Real-space correlation coefficients

#### A1.1. CC_box_: all grid points of the box are used

This is the most trivial correlation coefficient. It answers the question ‘how well does the atomic model reproduce the whole set of experimental data (three-dimensional map in cryo-EM)?’ Low values of CC_box_ do not necessarily mean that the model does not fit the map well around atomic positions, but may instead indicate that there are uninterpreted map features somewhere else in the ‘box’. The value of CC_box_ depends on the ‘box’ size and this is its major drawback; CC_box_ may be artificially high if the ‘box’ with a featureless map around the model is large.

#### A1.2. CC_mask_: grid points that belong to the molecular mask as defined by Jiang & Brünger (1994 ▸)

This mask is well established and routinely used in crystallography. It is independent of resolution, and CC_mask_ answers the question ‘how well does the available atomic model describe the part of the map around atomic centers (regardless of what is happening in other parts of the target map further away from the atomic model)?’. This is a reasonable question to ask at higher resolutions when atomic images are rather sharp. At lower resolution, the high map values are no longer situated on or near atomic centers and map comparison far from atomic centers becomes meaningful. The number of grid points inside the mask, *N*_mask_, is related to the volume of the molecule (this will be used below to define other types of CC).

#### A1.3. CC_vr_mask_: grid points inside a mask covering atomic images

The mask defined by Jiang & Brünger (1994 ▸) does not account for atomic density smearing owing to finite resolution and atomic displacement parameters. Therefore, one can envision a version of CC_mask_ where atomic radii account for these effects; we call this correlation coefficient CC_vr_mask_. In contrast to the previous mask built with prescribed unique radii, here atomic radii are chosen from atomic images corresponding to given atom type, map resolution and atomic displacement parameters. The simplest way to take the resolution dependence into account is to use an atom radius equal to the resolution value and to vary it around this value in order to maximize the CC. In this work, we applied a more formal procedure that does not involve an optimization step and is therefore easier to reproduce. We define the atomic radii from Fourier images of corresponding atoms (Urzhumtseva *et al.*, 2013 ▸; details are described in Appendix *D* [App appd]). The lower the resolution is, the larger the mask. We call the correlation coefficient calculated using such a mask CC_vr_mask_.

#### A1.4. CC_volume_: uses the top *N*_mask_ grid points with the highest values of the model map

This mask is composed from grid points with highest model map values ρ_mod_(**n**), *i.e.* those satisfying the condition ρ_mod_(**n**) ≥ μ_mod_. The value of μ_mod_ is chosen such that the number of selected grid points is equal to *N*_mask_ as defined above. This mask may exclude poorly defined and unreliable atoms such as loose side chains and loops (for which map values are low) and instead include points with a strong model density between the atoms.

#### A1.5. CC_peaks_: uses a union of the highest value grid points in the model and target maps

Here, the mask is similar to that used in the CC_volume_ calculation, except that instead of just choosing the highest *N*_mask_ points in the model map (the peaks), both the model map and the experimental map are considered and the union of the resulting masks is taken (Urzhumtsev *et al.*, 2014 ▸). Similar to CC_box_, and unlike CC_volume_, the CC_peaks_ value may be low if the model is incomplete.

Fig. 16 ▸ illustrates the regions for all five CCs defined above. For a model that interprets the map correctly, all five values are expected to be high.

One may note that CC_mask_ and CC_volume_ consider grid points only around atomic centers, while CC_box_ and CC_peaks_ consider points anywhere in the volume. Depending on the resolution and ADP, CC_vr_mask_ may belong to the first or to the second category. In practice, we did not meet a situation in which CC_vr_mask_ discriminated a model while it was accepted by other CCs (not shown) and thus we do not discuss it in the main text.

### A2. Fourier shell correlation (FSC) and soft mask

The FSC (see Rosenthal & Henderson, 2003 ▸ and references therein) is computed first by Fourier transformation of two maps to obtain two ‘boxes’ of Fourier map coefficients. The overall correlation between the two sets of Fourier coefficients is equal to CC_box_ and is therefore not very informative. More informative is to represent the Fourier correlation as a function of resolution. A curve of correlation *versus* the inverse of the resolution is then plotted. In practice, maps that are subject to FSC calculation are masked first (see, for example, Penczek, 2010 ▸; Pintilie *et al.*, 2016 ▸). While using a binary map (Jiang & Brünger, 1994 ▸) is problem-free for calculations in real space, it may be problematic for FSC calculations as sharp edges resulting from applying a binary map may introduce Fourier artifacts. Therefore, a ‘soft mask’ (see, for example, Rosenthal & Rubinstein, 2015 ▸; Pintilie *et al.*, 2016 ▸) that possesses a smooth boundary is desirable. Here, we calculate such a mask in the following way. Firstly, the binary mask (Jiang & Brünger, 1994 ▸) is calculated using inflated atomic radii, with the inflation radius *R*_smooth_ being set to the map resolution estimate, *d*_FSC_, from half-maps. This mask is then Fourier transformed into a box of corresponding Fourier map coefficients, which includes the *F*(0, 0, 0) term. Next, these Fourier coefficients are scaled by the resolution-dependent factor exp(−*Bs* ^2^/4), where *B* = 8π^2^ *R* ^2^_smooth_, and back Fourier transformed to yield the soft mask. Finally, a weighted CC_box_ is calculated using values of this soft mask as weight coefficients for the map values. Typically, for a pair of well correlated maps the FSC curve resembles an inverted sigmoid approaching 1 on the left side (low-resolution end) and falling off to zero on the right end of the plot (high resolution). For perfectly identical (up to a constant scale factor) maps the FSC is a straight horizontal line crossing the *y* axis at 1.

## Appendix B Resolution estimation from comparison of the experimental and model-based maps

When an atomic model corresponding to the experimental map is available, we can calculate a series of model maps at various resolutions and check which of them is the most similar to the experimental map. The resolution *d*_model_ of the model-calculated map that maximizes the correlation between the two maps may be considered as an estimate for the effective resolution cutoff of the experimental map.

In cryo-EM, atomic displacement parameters (ADPs, known also as *B* factors) are often undefined (all set to zero, for instance) or clearly nonsensical (see §[Sec sec3.4]3.4); also, it is customary in cryo-EM to apply various filters to the experimental map (blurring or sharpening, for example). It is therefore desirable to account for this by optimizing the overall isotropic ADP. As in crystallography (see, for example, Afonine, Grosse-Kunstleve *et al.*, 2013 ▸), the search for the optimal *B* value is performed in Fourier space by applying an overall isotropic, exponential, resolution-dependent scale factor to the map with the corresponding *B* value obtained by minimizing the residual

The overall scale factor *k* is irrelevant for CC calculations. Test calculations (not shown) confirm high robustness of this approach. Fig. 17 ▸ shows typical plots of CC_box_ as a function of trial resolution. In most cases the curve has a distinct peak maximum of correlation. However, we note that both decreasing resolution and increasing ADP values have a similar blurring effect on the images. As a consequence, for some data it may be difficult to distinguish between a higher value of resolution combined with a large ADP and a lower resolution combined with a smaller ADP.

## Appendix C Effective resolution cutoff of cryo-EM maps

Let ρ_tar_ be the initial cryo-EM map calculated on a rectangular grid inside an orthogonal parallelepiped which we consider to be a unit cell in space group *P*1. A Fourier transform of this map, considered as a periodic function, results in a ‘box’ of complex Fourier map coefficients, **F**_map_(**s**) = *F*_map_exp{φ_map_(**s**)}, **s** ∈ *S*_box_, which is an exact Fourier space equivalent of the corresponding real-space map, with the highest resolution coefficients being at the corners of the ‘box’. Let *d*_box_ be the highest resolution of the full ‘box’ of Fourier coefficients (the resolution of the coefficient that corresponds to one of the ‘box’ corners).

Starting from *d*_box_, we incrementally omit shells of high-resolution coefficients with a step of 0.01 Å in *d* spacing, and calculate the map ρ_cut_ using the remaining set, *S*_cut_. Next, we compare the map calculated using the truncated set of coefficients, *S*_cut_, with the initial map. This can be calculated efficiently using the reciprocal-space equivalent of the map correlation coefficient (Read, 1986 ▸; Lunin & Woolfson, 1993 ▸),

This function decreases with the resolution, and we note the resolution cutoff when (4) falls below some high enough critical value of correlation chosen in advance, which is the same for all structures. We consider that above this resolution the contribution of the Fourier coefficients is negligibly small and essentially does not change the map. Therefore, we accept this cutoff as the effective resolution cutoff of the data set corresponding to the initial map.

To determine the value of the correlation (4) that can be used to assign the resolution cutoff, we first selected the data sets for which we could calculate *d*_model_ (Appendix *B* [App appb]). For each of the selected data sets we then plotted (4) as a function of the resolution cutoff used to obtain ρ_cut_ (Fig. 18 ▸ *a*, black curve). We then sampled the CC values in the (0, 1) range to find a value at which the corresponding resolution cutoff *d*_CC_ would be closest to *d*_model_ (Fig. 18 ▸ *a*, red arrows). For each trial CC value we measured the similarity CC(*d*_model_, *d*_CC_) between *d*_model_ and *d*_CC_, calculated across all considered cases (Fig. 18 ▸ *b*). We found that CC = 0.99 maximizes the similarity and we refer to the corresponding resolution cutoff as *d*_99_ (Figs. 18 ▸ *a* and 18 ▸ *b*). Now, with this cutoff defined, the described procedure can be applied to any map regardless of whether an atomic model is present or not.

## Appendix D Determination of the atomic radius

For an atom with an isotropic scattering factor *f*(*s*) and an isotropic atomic displacement factor *B*, where *s* is the inverse resolution, *s* = 1/*d*, its image is spherically symmetric and can be described by its radial distribution ρ_d_(*r*), the image value as a function of the distance *r* to the atomic center. At a resolution cutoff *d*_high_, *i.e.* for *s* ≤ *s*_max_ = 1/*d*_high_, this function can be calculated as an integral

except for very small distances, *r* << 1, for which it is replaced by

These integrals can be calculated numerically using, for example, Simpson’s formula (see, for example, Atkinson, 1989 ▸). This calculation is very fast, giving an image of an isolated atom at a given resolution in a grid on *r* as fine as required.

For a given atomic image described by ρ_d_(*r*), different suggestions may be used to define its radius. Taking the first local minimum of the function or the zero closest to the origin are natural possibilities, but these values are numerically unstable when varying the resolution and *B* values. A more stable definition of the atomic radius refers to the definition of a critical (minimum) distance for an atomic image as a distance to the inflection point of ρ_d_(*r*) closest to the origin (Urzhumtseva *et al.*, 2013 ▸). The atomic radius is logically defined as twice this minimum distance (Fig. 19 ▸ *a*).

An additional advantage of our definition of the atomic radius is that while the atomic shape is different for different types of macromolecular atoms (C, N, O, P and S), the critical distance is similar for all of them (Urzhumtseva *et al.*, 2013 ▸) and therefore its knowledge for a C atom for a set of different *B* values and different resolutions is sufficient to obtain an interpolated radius value for each individual atom at any resolution and *B* factor. Note that as expected the radius increases with resolution and with the *B* value (Fig. 19 ▸ *b*). For particular types of atoms, for example heavy atoms, it is trivial to repeat the curve calculations as described above.

## Appendix E Model–map correlation coefficient (CC) values

The values of the correlation coefficient range between −1 for perfectly anticorrelated data and +1 for perfectly correlated data; 0 represents uncorrelated data. In structure-solution methods such as crystallography or cryo-EM, an accepted rule of thumb is to think of CC > 0.7 as a good fit and CC < 0.5 as a poor fit. Obviously, this is very arbitrary and is highly dependent on the problem and on the personal choice of the researcher. To facilitate the interpretation of CC values, we provide a relationship between CC and the coordinate error of an atomic model by doing the following. We place a model into a *P*1 box, set ADP values to a given value and calculate a map (M) of specified resolution from such model. We then subject this model to a molecular-dynamics simulation and calculate CC_mask_ values between M and maps calculated for models along the simulation trajectory. We record this CC along with the corresponding r.m.s. deviation between the original model and the intermediate model. The MD simulation continues until the CC reaches zero. This defines the CC as a function of model deviation. The entire calculation is repeated for several resolutions and ADP values. Each calculation was performed for two very different models: a protein and an RNA molecule. Fig. 20 ▸ indicates that a model–map correlation of 0.5 corresponds to a range of model errors from about 1.5 to 3.0 Å, and a correlation of 0.7 corresponds to model errors of 0.9–2.2 Å. Also, note that this result is relatively model-independent. Throughout the article we use these correlation values, 0.5 and 0.7, as reference values.
